# Different bimodal neuromodulation settings reduce tinnitus symptoms in a large randomized trial

**DOI:** 10.1038/s41598-022-13875-x

**Published:** 2022-06-30

**Authors:** Brendan Conlon, Caroline Hamilton, Emma Meade, Sook Ling Leong, Ciara O Connor, Berthold Langguth, Sven Vanneste, Deborah A. Hall, Stephen Hughes, Hubert H. Lim

**Affiliations:** 1 Neuromod Devices Limited, Dublin, D08 R2YP Ireland; 2 School of Medicine, Trinity College Dublin, Dublin, D02 R590 Ireland; 3 Department of Otolaryngology, St. James’s Hospital, Dublin, D08 NHY1 Ireland; 4 Trinity Institute for Neuroscience and Global Brain Health Institute, School of Psychology, Trinity College Dublin, Dublin, D02 PN40 Ireland; 5 Department of Psychiatry and Psychotherapy, University of Regensburg, 93053 Regensburg, Germany; 6 Interdisciplinary Tinnitus Center of the University of Regensburg, 93053 Regensburg, Germany; 7 Lab for Clinical and Integrative Neuroscience, School of Behavioral and Brain Sciences, The University of Texas at Dallas, Dallas, 75080 USA; 8 National Institute for Health Research Nottingham Biomedical Research Centre, Nottingham, NG7 2UH UK; 9 Hearing Sciences, Mental Health and Clinical Neurosciences, University of Nottingham, Nottingham, NG7 2RD UK; 10 School of Social Sciences, Heriot-Watt University Malaysia, 62200 Wilayah Persekutuan Putrajaya, Malaysia; 11 Department of Otolaryngology-Head and Neck Surgery, University of Minnesota, Minneapolis, 55455 USA; 12 Department of Biomedical Engineering, University of Minnesota, 312 Church Street S.E., NHH 7-105, Minneapolis, MN 55455 USA

**Keywords:** Biotechnology, Neuroscience, Engineering

## Abstract

More than 10% of the population suffers from tinnitus, which is a phantom auditory condition that is coded within the brain. A new neuromodulation approach to treat tinnitus has emerged that combines sound with electrical stimulation of somatosensory pathways, supported by multiple animal studies demonstrating that bimodal stimulation can elicit extensive neural plasticity within the auditory brain. More recently, in a large-scale clinical trial, bimodal neuromodulation combining sound and tongue stimulation drove significant reductions in tinnitus symptom severity during the first 6 weeks of treatment, followed by diminishing improvements during the second 6 weeks of treatment. The primary objective of the large-scale randomized and double-blinded study presented in this paper was to determine if background wideband noise as used in the previous clinical trial was necessary for bimodal treatment efficacy. An additional objective was to determine if adjusting the parameter settings after 6 weeks of treatment could overcome treatment habituation effects observed in the previous study. The primary endpoint at 6-weeks involved within-arm and between-arm comparisons for two treatment arms with different bimodal neuromodulation settings based on two widely used and validated outcome instruments, Tinnitus Handicap Inventory and Tinnitus Functional Index. Both treatment arms exhibited a statistically significant reduction in tinnitus symptoms during the first 6-weeks, which was further reduced significantly during the second 6-weeks by changing the parameter settings (Cohen’s *d* effect size for full treatment period per arm and outcome measure ranged from − 0.7 to − 1.4). There were no significant differences between arms, in which tongue stimulation combined with only pure tones and without background wideband noise was sufficient to reduce tinnitus symptoms. These therapeutic effects were sustained up to 12 months after the treatment ended. The study included two additional exploratory arms, including one arm that presented only sound stimuli during the first 6 weeks of treatment and bimodal stimulation in the second 6 weeks of treatment. This arm revealed the criticality of combining tongue stimulation with sound for treatment efficacy. Overall, there were no treatment-related serious adverse events and a high compliance rate (83.8%) with 70.3% of participants indicating benefit. The discovery that adjusting stimulation parameters overcomes previously observed treatment habituation can be used to drive greater therapeutic effects and opens up new opportunities for optimizing stimuli and enhancing clinical outcomes for tinnitus patients with bimodal neuromodulation.

## Introduction

Tinnitus is a phantom auditory sensation that is coded within the brain^1^, and can be bothersome or debilitating for 10–15% of the population^1–4^. It continues to be a major health issue in our society with limited treatment options^5,6^. Encouragingly, there has been a recent convergence of findings across multiple animal and human studies demonstrating that bimodal neuromodulation combining sound with electrical stimulation of peripheral nerves, including vagus, trigeminal and other somatosensory nerves, can drive neural plasticity relevant for tinnitus treatment and significantly improve tinnitus symptoms^7–16^. Previous animal studies have shown that bimodal stimulation combining electrical body stimulation combined with pure tones or wideband noise can alter brain activity within auditory regions associated with tinnitus^7,8,13–15^. These studies demonstrated that electrical stimulation of the vagus or trigeminal nerves or the surface of different body regions in animals (e.g., tongue, face, neck and ear) can alter brain patterns relevant for tinnitus treatment and reduce tinnitus symptom severity in human patients. Furthermore, different patterns of stimuli appear to be effective. Bimodal neuromodulation comprising pure tones or wideband noise drive significant neural plasticity in the brainstem, midbrain or cortex along the auditory pathway^7,8,10,13–16^. Different timing schemes between sound stimulation and somatosensory nerve stimulation have also been shown to reduce tinnitus symptoms, in which interstimulus delays can vary up to hundreds of milliseconds^7,9,11^.

**Figure 1:**
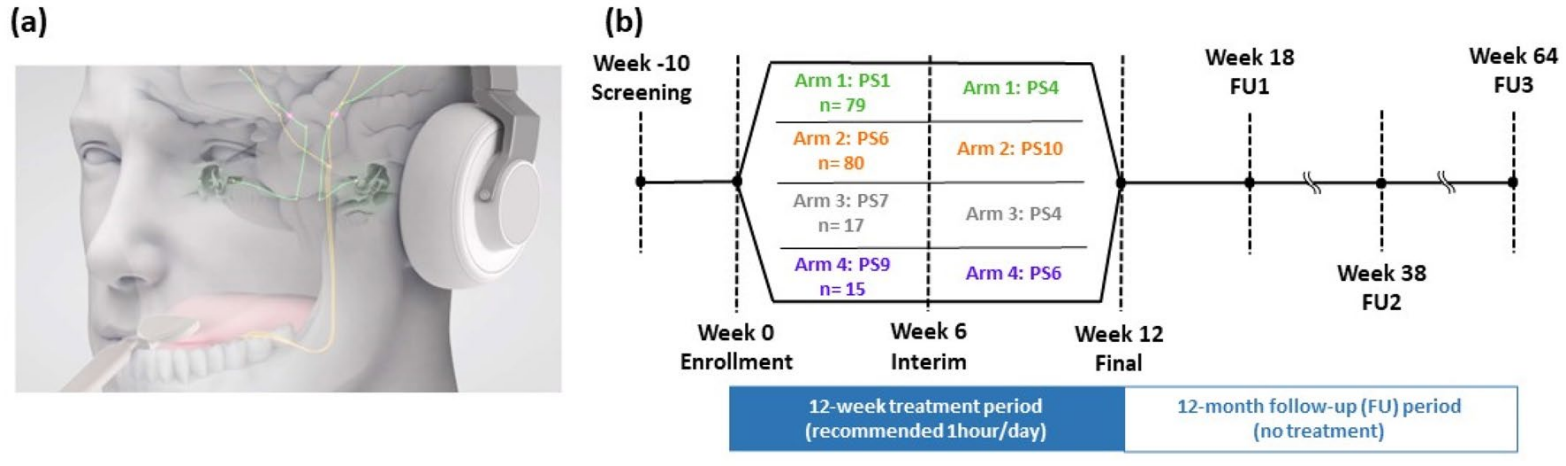
Timeline of different parameter settings for tinnitus treatment in the TENT-A2 study. (**a**) The Lenire bimodal neuromodulation device developed by Neuromod Devices is shown (CE-marked Class IIa; previously referred to as MBT in the study). Sound stimulation is delivered through wireless headphones and electrical stimulation is presented to the anterior-dorsal surface of the tongue via a 32-site electrode array. Both stimulation approaches are controlled using a battery-powered controller. Bimodal stimulation activates auditory and somatosensory pathways, as well as converging centers in auditory, limbic and attentional brain regions implicated in tinnitus. (**b**) Timeline of four treatment arms with different stimulation parameters over time (see Supplementary Table S1 for detailed description). Arm 1 and arm 2 are included for the primary endpoint analyses. Arm 3 and arm 4 are included for additional analyses. Evaluations of device safety and efficacy were conducted at interim and final visits with post-treatment follow-ups planned at weeks 18, 38 and 64 from enrollment.

In 2020, results from a large randomized and blinded clinical trial for bimodal neuromodulation (TENT-A1 study; clinicaltrials.gov: NCT02669069) with 326 enrolled participants supported the safety and efficacy of combined sound and tongue stimulation therapy using the Lenire device (Fig. 1a; CE-marked Class IIa medical device; Neuromod Devices, Ireland)^11^. The TENT-A1 study showed that bimodal neuromodulation reduced tinnitus symptom severity scores in more than 80% of participants during the 12-week treatment period that could last for 12 months after the treatment ended. The scores were based on two widely used and validated outcome instruments: Tinnitus Handicap Inventory (THI) and Tinnitus Functional Index (TFI)^17–20^. About two-thirds of the participants also reported that they had benefitted from the tinnitus treatment based on exit interview questions. These outcomes were consistent across all three treatment arms in the study that included different parameter settings, and there were no significant differences between arms. As detailed in the published TENT-A1 manuscript^11^, “arm 1 (PS1 setting) consisted of synchronized sound and tongue stimulation. A pure tone (ranging approximately from 500 to 8000 Hz, with repetition period of about 80 ms) was presented at the same time that a train of pulses was presented to a specific location on the tongue. The stimulation location on the tongue array was fixed for a given pure tone frequency; this resulted in a fixed tone-to-tongue spatial mapping. Arm 2 (PS2 setting) used the same auditory stimuli as PS1 but had short interstimulus delays that varied in the range of 30–50 ms across each stimulus presentation and the tone-to-tongue spatial mapping was randomized across stimulus presentations. Arm 3 (PS3 setting) used lower frequency tones (approximately 100 to 500 Hz) with longer interstimulus delays (550–950 ms) than used in PS2.” All three parameter settings included background wideband noise and applied supra-threshold intensities for sound and tongue stimulation. These settings were fixed across the 12-week treatment period.

Two interesting observations from that previous TENT-A1 study^11^ have raised further questions about which stimulus components and parameters are necessary and sufficient for treatment success. The first observation was that all three combinations of parameter settings significantly reduced tinnitus symptom severity, despite different tone frequencies, tongue stimulus patterns and interstimulus delays. Given that all three combinations included background wideband noise, this raises the important question as to whether or not the noise feature was necessary for bimodal treatment to be effective. The second observation was that significant improvements in tinnitus symptom severity occurred primarily within the first 6 weeks of treatment, with diminishing improvements during the second 6 weeks of treatment. This raises the question as to whether adjusting the parameter settings during treatment could overcome the habituation effects and drive further therapeutic benefits. The motivating reasons for the current study, referred to as TENT-A2 (Treatment Evaluation of Neuromodulation for Tinnitus—Stage 2; clinicaltrials.gov: NCT03530306), were to investigate and answer these two questions, as well as to confirm the safety and efficacy results observed in the TENT-A1 study. The study design was double-blinded because all participants were informed that they were receiving an active treatment and neither they nor the participant-facing investigators could ascertain which treatment arm would provide the greatest therapeutic benefit.

TENT-A2 was statistically powered (arm 1 and arm 2 in Fig. 1b) to test the first question about whether the wideband noise component of the sound stimulus was necessary for therapeutic benefit at the 6-week endpoint. The noise parameter was present in the parameter settings for arm 1 and absent for arm 2 (see Supplementary Table S1 for a description of parameter settings). Additional analysis assessed the second question about the effect of adjusting the sound and tongue stimulus parameters at the 6-week (interim) visit. In arm 1, the pure tone presentation and tongue stimulation were changed from being synchronous to having a varied interstimulus delay with the location of stimulation on the tongue randomized across stimuli (i.e., PS1–PS4). In arm 2, the sound stimulus changed from pure tone stimuli to noise bursts with a different range of interstimulus delays (i.e., PS6 to PS10). Comparisons at multiple time points depicted in Fig. 1b, including follow-up visits, were allocated to exploratory analyses.

The study design also included two exploratory arms (arm 3 and arm 4) to investigate the contribution of different sound and tongue stimulus components on therapeutic outcomes, without an expectation of hypothesis testing. The first 6-week period in arm 3 investigated the efficacy of a stimulation regime using four simultaneous tones instead of single or dual tones as in the other arms. In arm 4, the first 6-week period comprised a sound-only condition (without tongue stimulation), after which tongue stimulation was added in at the interim visit. Further details and rationale for the parameter settings used in each arm are provided in a previously published protocol paper^21^ with a summary of the parameter settings in Supplementary Table S1.

The clinical site for the study was the Wellcome Trust-HRB Clinical Research Facility at St. James’s Hospital in Dublin, Ireland. The study protocol was independently reviewed and approved by the Research Ethics Committees of the Tallaght University Hospital-St James’s Hospital (Reference: 2018-03-List-9). The study was registered at clinicaltrials.gov (NCT03530306). All methods were carried out in accordance with relevant guidelines and regulations. Participants that were enrolled into the study were fitted with the Lenire device and instructed to use it for one hour per day for 12 weeks, with three follow-up visits up to 12 months after the treatment ended (Fig. 1b). As in the TENT-A1 study, THI and TFI were used for the outcome measures of the TENT-A2 study. Safety, acceptability, and efficacy results for the Lenire device for the full cohort of participants are presented in this paper. Other planned analyses of subgroups, including patients with co-morbid hyperacusis and high tinnitus symptom severity groups, and additional exploratory investigations of different types of tinnitus outcome measures will be presented in a subsequent publication. Further details on the study design and analysis plan are provided in a previously published protocol paper^21^.

## Results

### Characteristics and summary of study participants

**Figure 2:**
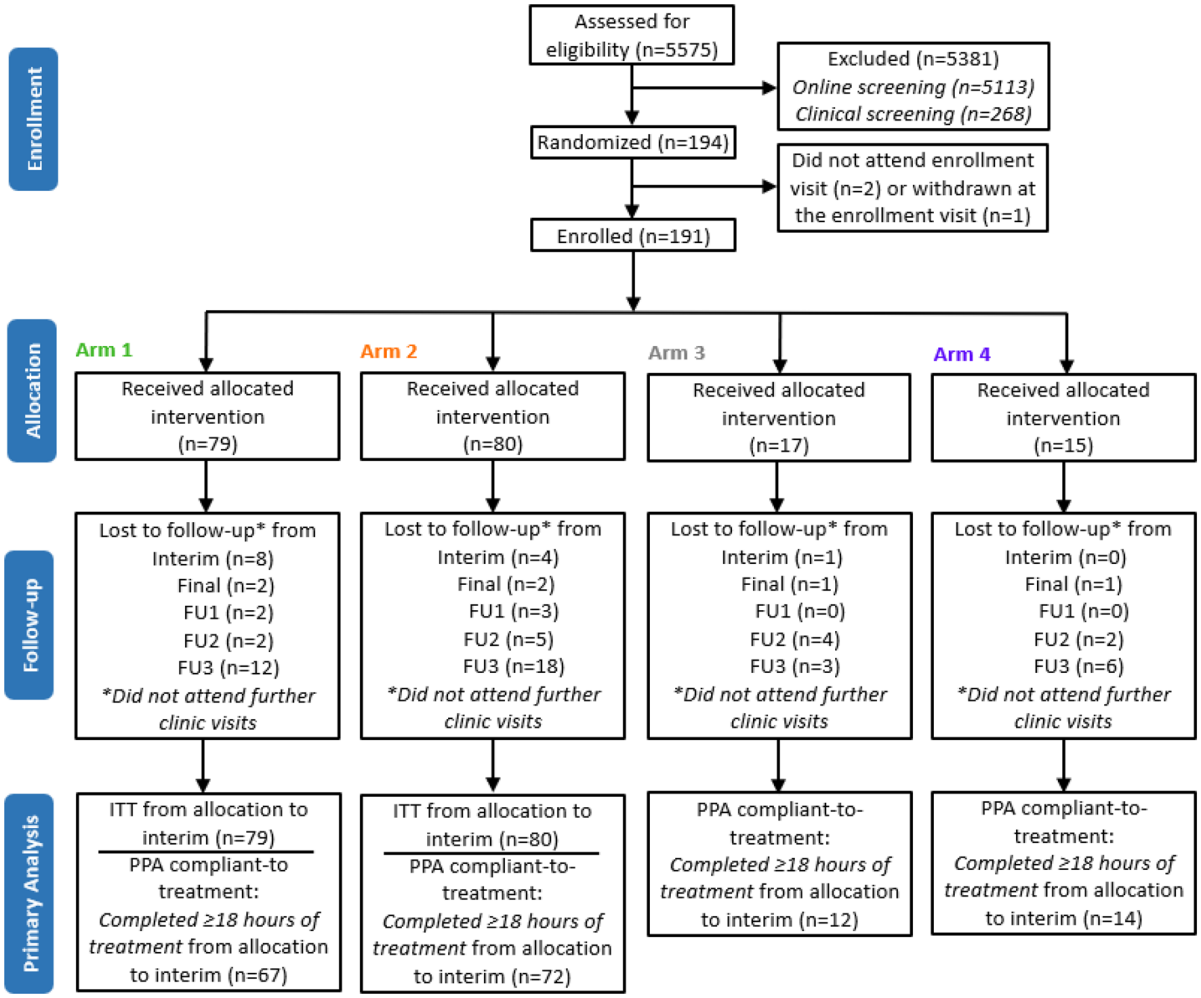
Participant flow diagram. For the primary endpoint analyses, within-arm comparisons from start of treatment to the 6-week assessment for THI and TFI were performed with per-protocol analysis (PPA) for those who were compliant to treatment (at least 18 h of device usage over 6 weeks of treatment period). Only arm 1 was included for the within-arm primary endpoint analyses whereas the other arms were included as additional analyses. Between-arm comparison was performed only between arm 1 and arm 2 for the primary endpoint analyses for changes in THI scores from start of treatment to the 6-week assessment using intention-to-treat (ITT) with imputation for missing values described in the Methods. Additional exploratory analyses for THI and TFI were performed at other time points during treatment and up to week 64 (12 months post-treatment) that are presented in this paper, with compliance and participant number information provided where appropriate for those analyses. Follow-up (FU) visits are depicted in Fig. 1.

Study participants were recruited to the clinical trial using radio advertising and directed to an online eligibility assessment. Eligibility was initially assessed through a set of general pre-screening questions to manage the large number of candidates expected to respond to the advertising^22^. Of these potential participants, 462 signed an informed consent and attended a clinical screening visit. On the basis of the inclusion and exclusion criteria, 194 participants were randomized into the four treatment arms (Fig. 2). Two of those individuals did not attend the enrollment visit while one participant was withdrawn at the enrollment visit prior to receiving the device by the study investigators, resulting in a total of 191 individuals completing enrollment and device fitting.

As shown in Table 1, there were no significant differences between arm 1 and arm 2 (*P* > 0.05; relevant for primary endpoint analyses) pertaining to several characteristics and stratification categories. There was a high retention rate of participants with 89.9% and 95.0% of enrolled participants returning to the 6-week (interim) visit for arm 1 and arm 2, respectively. Across all four arms there was also a high retention rate of participants of 93.2% at the 6-week visit and 90.6% at the 12-week visit when the treatment ended. The high retention rate in this study is aligned with the high treatment compliance rate at the 6-week and 12-week assessments of 86.4% and 83.8%, respectively (Supplementary Fig. S2). Compliance at 6-weeks was defined as device usage of at least 18 h in the first 6 weeks of treatment while compliance at 12-weeks was defined as device usage of at least 36 h during the 12 weeks of treatment; these criteria were based on findings from a previous large-scale tinnitus treatment study using the Lenire device^11^. The device logged the time and date of daily usage by each participant. There were no significant differences in the compliance rate or number of participants between arm 1 and arm 2 that are relevant for the primary endpoint analyses (*P* > 0.05). Participants attended their visits at the intended time points during the treatment period, as well as the 12-month follow-up visit (Week 64), as shown in Supplementary Fig. S3. Due to scheduling conflicts, there was an average delay of one week for the Week 18 visit (6-week follow-up) and an average shift of three weeks earlier for the Week 38 visit (6-month follow-up). These shifts in post-treatment assessment dates did not affect the primary endpoint analyses of the study, which were based on only the first 6 weeks of treatment.

**Table 1:** Characteristics of enrolled participants in each arm with key numbers for each visit.

Characteristics	Units	Full cohort	Arm 1	Arm 2	Arm 3	Arm 4	P-value
Total participants enrolled (device fitted)	# Participants	191	79	80	17	15	1.000
Gender: male	# Participants (% enrolled)	128	54 (68.4%)	58 (72.5%)	8 (47.1%)	8 (53.3%)	0.894
Gender: female	# Participants (% enrolled)	63	25 (31.6%)	22 (27.5%)	9 (52.9%)	7 (46.7%)	0.838
Age at screening	Years [mean (SD)]	50.3 (10.7)	51.7 (10.1)	49.0 (11.0)	49.5 (10.2)	50.9 (13.0)	0.122
Tinnitus duration at screening	Years [mean (SD)]	4.1 (2.8)	3.9 (2.7)	4.3 (3.0)	5.3 (3.0)	3.3 (2.6)	0.418
THI at screening	Points [mean (SD)]	54.4 (13.7)	54.8 (13.9)	53.9 (12.9)	50.7 (10.3)	59.1 (18.8)	0.695
Mean hearing loss (0.25-8kHz) at screening	dB HL [mean (SD)]	21.4 (11.2)	21.5 (10.9)	22.2 (11.0)	18.0 (10.2)	21.3 (15.2)	0.699
Attended interim visit	# Participants (% of enrolled)	178 (93.2%)	71 (89.9%)	76 (95.0%)	16 (94.1%)	15 (100.0%)	0.816
Attended final visit	# Participants (% of enrolled)	173 (90.6%)	70 (88.6%)	74 (92.5%)	15 (88.2%)	14 (93.3%)	0.906
Attended FU1 visit	# Participants (% of enrolled)	165 (86.4%)	66 (83.5%)	70 (87.5%)	15 (88.2%)	14 (93.3%)	0.903
Attended FU2 visit	# Participants (% of enrolled)	150 (78.3%)	64 (81.0%)	65 (81.3%)	9 (52.9%)	12 (80.0%)	1.000
Attended FU3 visit	# Participants (% of enrolled)	116 (60.7%)	54 (68.4%)	48 (60.0%)	8 (47.1%)	6 (40.0%)	0.779
**Stratification**							
Hyperacusis (LDL < 70dB SL at 500Hz)	# Participants (% of enrolled)	61 (31.9%)	25 (31.7%)	25 (31.3%)	5 (29.4%)	6 (40.0%)	1.000
Hyperacusis (LDL < 60dB SL at 500Hz)	# Participants (% of enrolled)	23 (12.0%)	10 (12.7%)	10 (12.5%)	1 (5.9%)	2 (13.3%)	1.000
High THI (THI > 56 points)	# Participants (% of enrolled)	72 (37.7%)	30 (38.0%)	30 (37.5%)	5 (29.4%)	7 (46.7%)	1.000
Unilateral tinnitus	# Participants (% of enrolled)	51 (26.7%)	20 (25.3%)	23 (28.8%)	4 (23.5%)	4 (26.7%)	0.831

dB HL, decibel hearing level; dB SL, decibel sensation level (equals dB HL minus hearing threshold level of sound stimulus); FU, follow-up; LDL, loudness discomfort level. A description of stratification categories is provided in the Methods. P-values were calculated for comparing arm 1 and arm 2 (i.e., arms used for primary endpoint analyses) using Fisher’s exact test for count variables or a linear regression for continuous variables. Participants were stratified into different arms based on hyperacusis level, THI severity and presence of a unilateral tinnitus.

### Background wideband noise stimulation is not necessary for achieving bimodal treatment efficacy

In arm 1, PS1 was provided during the first 6 weeks of treatment. PS1 consisted of pure tone bursts temporally and spatially synchronized with electrical pulses presented to different locations on the surface of the tongue, together with background wideband noise. PS6 in arm 2 consisted of only pure tone bursts paired with spatially randomized tongue stimulation with varying interstimulus delays (700–800 ms) and without any background noise. We observed in the previous TENT-A1 study^11^ that a synchronized sound and tongue stimulation paradigm or a spatially-temporally varying sound and tongue stimulation paradigm both achieved significant improvements in tinnitus symptoms when including wideband noise, and there were no significant differences between paradigms. Thus, both types of stimulation paradigms were included in this TENT-A2 study, except that arm 2 did not include wideband noise. Both sound and tongue stimuli were presented at supra-threshold intensities. A description of the parameter settings used in arm 1 and arm 2 are presented in Supplementary Table S1.

**Figure 3:**
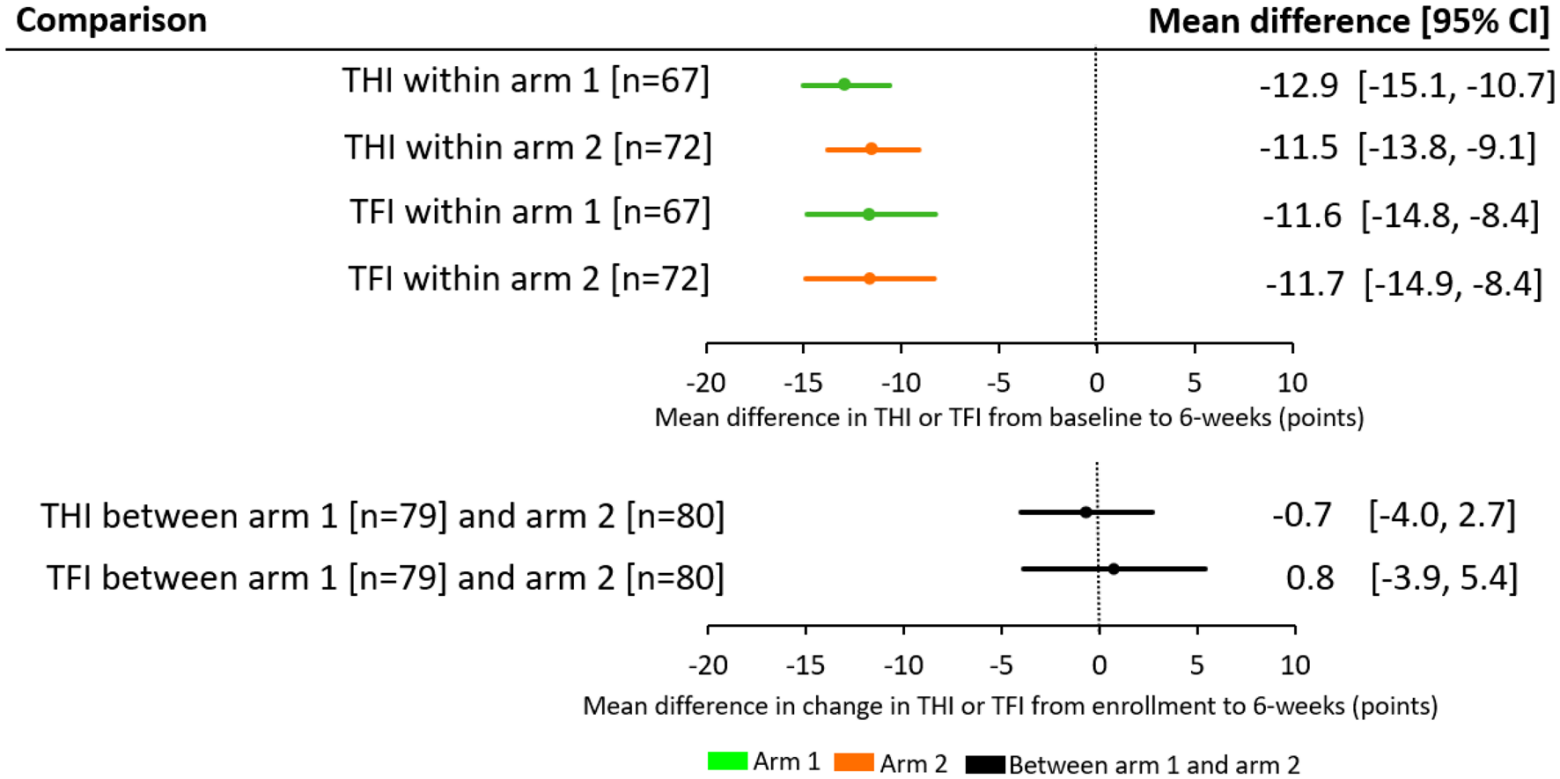
Within-arm and between-arm analyses for primary endpoints at 6-weeks. The mean differences in THI and TFI scores across participants for arm 1 and arm 2 from baseline to 6-week assessment for the within-arm cases, or the mean differences between arm 1 and arm 2 for the change in scores from enrollment to 6-week assessment for the between-arm cases are plotted with the ± 95% CI. Within-arm analyses were based on per-protocol analysis that included treatment-compliant participants (≥ 18 h treatment at 6-weeks) with baseline and 6-week scores. The baseline score corresponds to the average of the scores at screening and enrollment visits. Within-arm analyses for arm 1 and arm 2 showed a highly statistically significant reduction in THI and TFI scores (i.e., improvement in tinnitus symptom severity) based on paired two-tailed t-tests (*P* < 0.0001; 95% CI does not overlap the vertical line at zero). Between-arm analyses were conducted using an intention-to-treat analysis with changes in THI or TFI scores from the enrollment to 6-weeks assessment and tested with multiple regression using enrollment score as a covariate. Missing data were imputed using the Markov chain Monte Carlo multiple imputation method (further details are provided in the Methods), which leads to n values that match the enrolled numbers for each arm. There was no significant difference between arm 1 and arm 2 (*P* > 0.05; 95% CI crosses vertical line at zero). Note that primary endpoint analyses included within-arm changes for THI and TFI for arm 1 and between-arm differences for arm 1 and arm 2 only for THI, but that the other within-arm and between-arm comparisons are presented in this figure for completeness. Parameter settings (see Supplementary Table S1 for details): arm 1—PS1 (synchronized bimodal stimulation, includes pure tones and background noise stimuli); arm 2—PS6 (includes only pure tones with no background noise stimuli and with varied interstimulus delays in the range of 700–800 ms).

At the 6-week assessment, arm 1 and arm 2 exhibited highly significant reductions in THI and TFI scores relative to their baseline scores (Fig. 3; *P* < 0.0001, paired two-tailed t-tests; adjusted significance level for multiple comparisons is 0.0025 described in Conlon et al.^21^). These improvements in tinnitus symptom severity correspond to an average reduction in THI score of 12.9 and 11.5 points for arm 1 and arm 2, respectively, and in TFI score of 11.6 and 11.7 points, respectively (Fig. 3). For Cohen’s *d*^23^, arm 1 exhibited an effect size (with confidence interval, CI) of − 0.8 [95% CI: − 1.0, − 0.6] for THI and − 0.6 [95% CI: − 0.7, − 0.4] for TFI, while arm 2 exhibited an effect size of − 0.8 [95% CI: − 1.0, − 0.6] for THI and − 0.7 [95% CI: − 0.9, − 0.5] for TFI. A *d* value of 0.5 is considered a moderate effect size while a *d* value of 0.8 is considered a large effect size, indicating that bimodal stimulation drives a moderate to large reduction in tinnitus symptom severity within a 6-week treatment period. Although the within-arm primary endpoints were achieved in this study, there was no significant difference between arm 1 and arm 2 (*P* > 0.0275; adjusted significance level for multiple comparisons described in Conlon et al.^21^). Although this null difference does not reveal which parameter setting is superior (i.e., arm 1 versus arm 2), the within-arm results confirm that the background wideband noise component is not necessary for achieving significant reductions in tinnitus symptom severity with bimodal stimulation. As observed in the previous TENT-A1 study^11^, synchronous bimodal stimulation or stimulation with interstimulus delays of several hundred milliseconds, as well as different tone-to-tongue mapping strategies, are all sufficient to drive significant reductions in tinnitus symptom severity.

### Therapeutic benefit is boosted by adjusting parameter settings and is sustained long term

**Figure 4:**
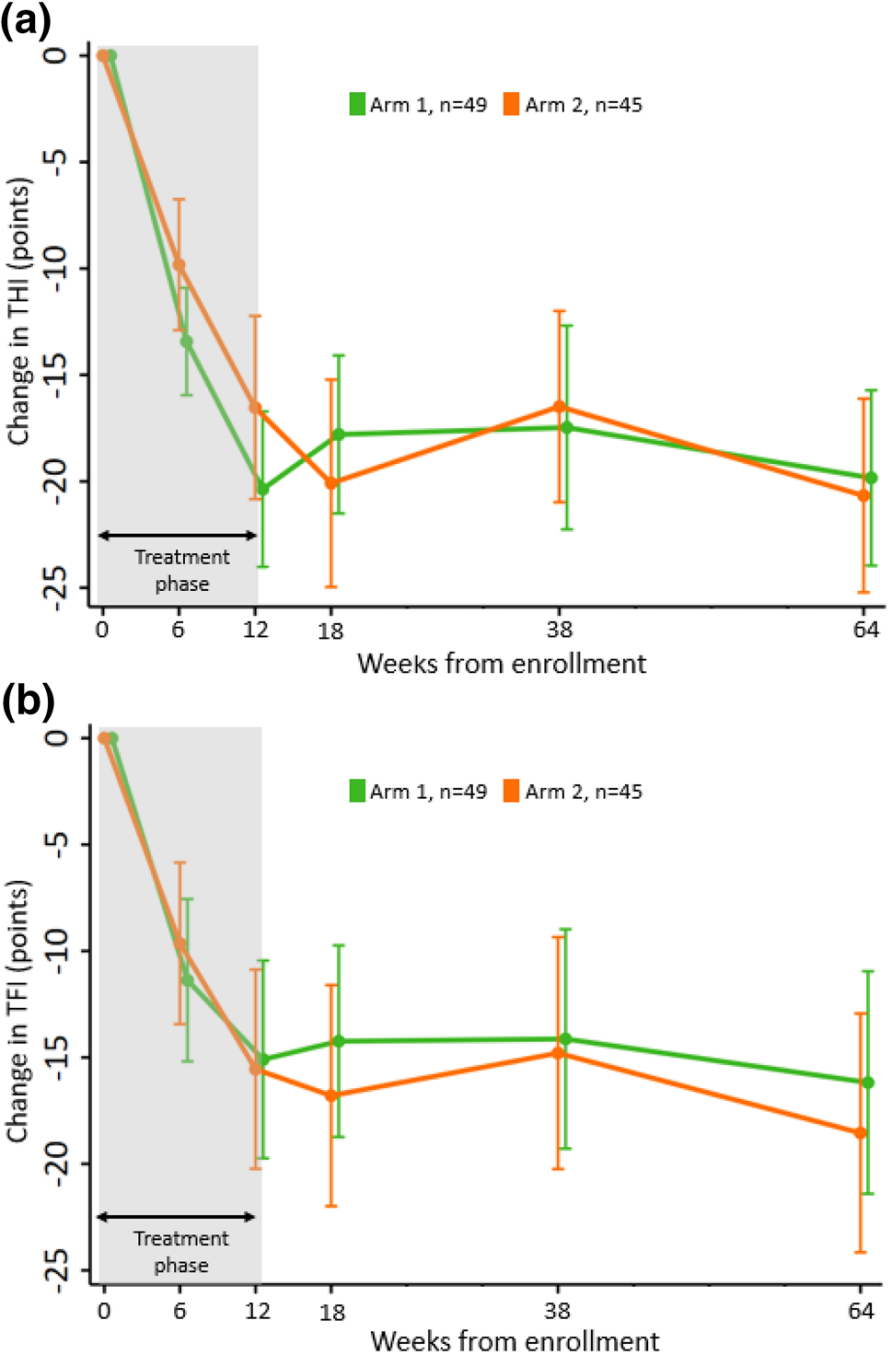
Long-term clinical efficacy of bimodal neuromodulation. Change in THI score (**a**) or TFI score (**b**) from baseline to the different time points are plotted up to the 12-month post-treatment visit (Week 64) for participants who were treatment-compliant (≥ 18 h treatment at interim and ≥ 36 h treatment at final) and attended all visits. Mean change values and 95% CIs are plotted for each arm. All data points with CIs are substantially below the zero line, supporting that bimodal neuromodulation with sound and tongue stimulation achieves significant reduction in tinnitus symptoms that is sustained for 12 months after treatment ended. For clearer visualization, data points and error bars were jittered in time.

**Figure 5:**
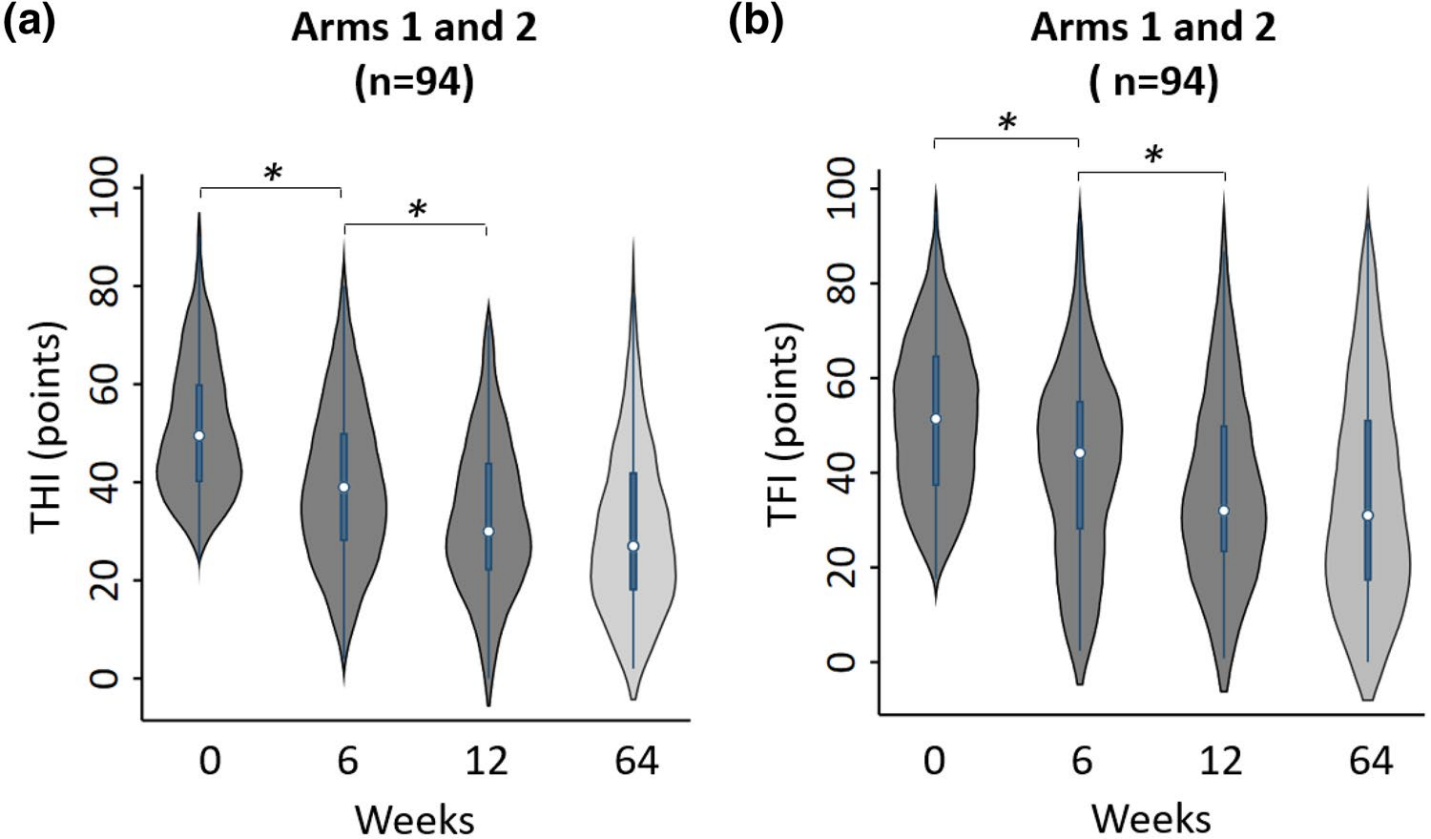
Additional reduction in tinnitus symptoms with adjustment of parameter settings at the midway point during treatment. Absolute THI or TFI scores (distribution of values as violin plots) are presented at different time points during the treatment period at Week 0 (baseline), Week 6 (interim), Week 12 (final) and Week 64 (12-month post-treatment) for pooled data across arm 1 and arm 2 for THI (**a**) and TFI (**b**). Data is included for participants who were treatment-compliant (≥ 18 h treatment at interim and ≥ 36 h treatment at final) and correspond to the same data shown in Fig. 4. Different stimulus combinations were implemented in the first versus second 6-week period. Asterisks correspond to significant reductions in THI or TFI scores based on a Wilcoxon signed-rank test (*P* < 0.05). Significant P-values accounting for multiple comparisons based on the Bonferroni correction are labeled with asterisks in order from left to right in each plot: (**a**) *P* < 0.00001, *P* < 0.00001; (**b**) *P* < 0.00001, *P* = 0.0047.

The study was designed to track changes in tinnitus symptom severity at multiple time points during treatment and up to 12 months after the end of treatment. As shown in Fig. 4, there was a large reduction in tinnitus symptoms during the first 6 weeks of treatment, which continued to decrease during the second 6 weeks of treatment after the stimulation setting was changed from PS1 to PS4 in arm 1 and PS6 to PS10 in arm 2 (Fig. 4). This pattern of treatment outcome differs from the previous TENT-A1 study in which improvements diminished during the second 6 weeks of treatment, when presenting the same stimulation setting for the entire 12-week treatment period^11^. As further shown in Fig. 5, when adjusting the stimulation setting midway through the treatment period, there were significant reductions in THI and TFI scores during the first 6 weeks of treatment and also during the second 6 weeks of treatment. A significant reduction in THI or TFI score was not observed during the second 6 weeks of treatment in the previous TENT-A1 study (Supplementary Fig. S4). These contrasting findings were consistently observed across individual treatment arms (Supplementary Figs. S5 and S6). At the end of the 12-week treatment period for the TENT-A2 study, there was an average reduction across both arms of 18.5 points for THI and 15.3 points for TFI relative to the baseline scores. The improvements in tinnitus symptom severity were sustained for 12 months after treatment was withdrawn, resulting in an average long-term reduction of 20.2 points for THI and 17.3 points for TFI. For the TENT-A1 study (see Fig. 5 in that study paper^11^), there was an average reduction across arms of 12.6 points for THI and 12.0 points for TFI with a long-term reduction of 12.8 points for THI and 14.4 points for TFI, which are less than the improvements observed in the TENT-A2 study.

### Different bimodal parameter settings improve tinnitus symptoms over time

For the primary endpoints, comparisons were performed for within-arm and between-arm changes in THI and TFI for arm 1 and arm 2. From those analyses, we determined that significant therapeutic effects are achievable when providing bimodal neuromodulation using tongue stimulation combined with only pure tones (i.e., without requiring background noise stimuli) and with varying interstimulus delays of up to 800 ms. A fixed tone-to-tongue spatial mapping was also not essential for improving tinnitus symptoms with bimodal neuromodulation.

Including two exploratory arms (arm 3 and arm 4) in the study design enabled two additional questions regarding the therapeutic efficacy of different stimulation parameters to be further investigated. The first explored whether simultaneously presented tones are sufficient to drive therapeutic effects. In the first half of the 12-week treatment period, PS7 was used in arm 3, which consisted of four inharmonic-related tones instead of a single or harmonically-related dual tone as occurred for the other arms. The second question investigated the contribution of the tongue stimulation component on treatment efficacy. Arm 4 comprised the PS9 parameter setting, which included only the sound component of PS6 presented in arm 2 and excluded tongue stimulation. These exploratory arms serve to identify interesting research hypotheses to be tested in future studies.

**Figure 6:**
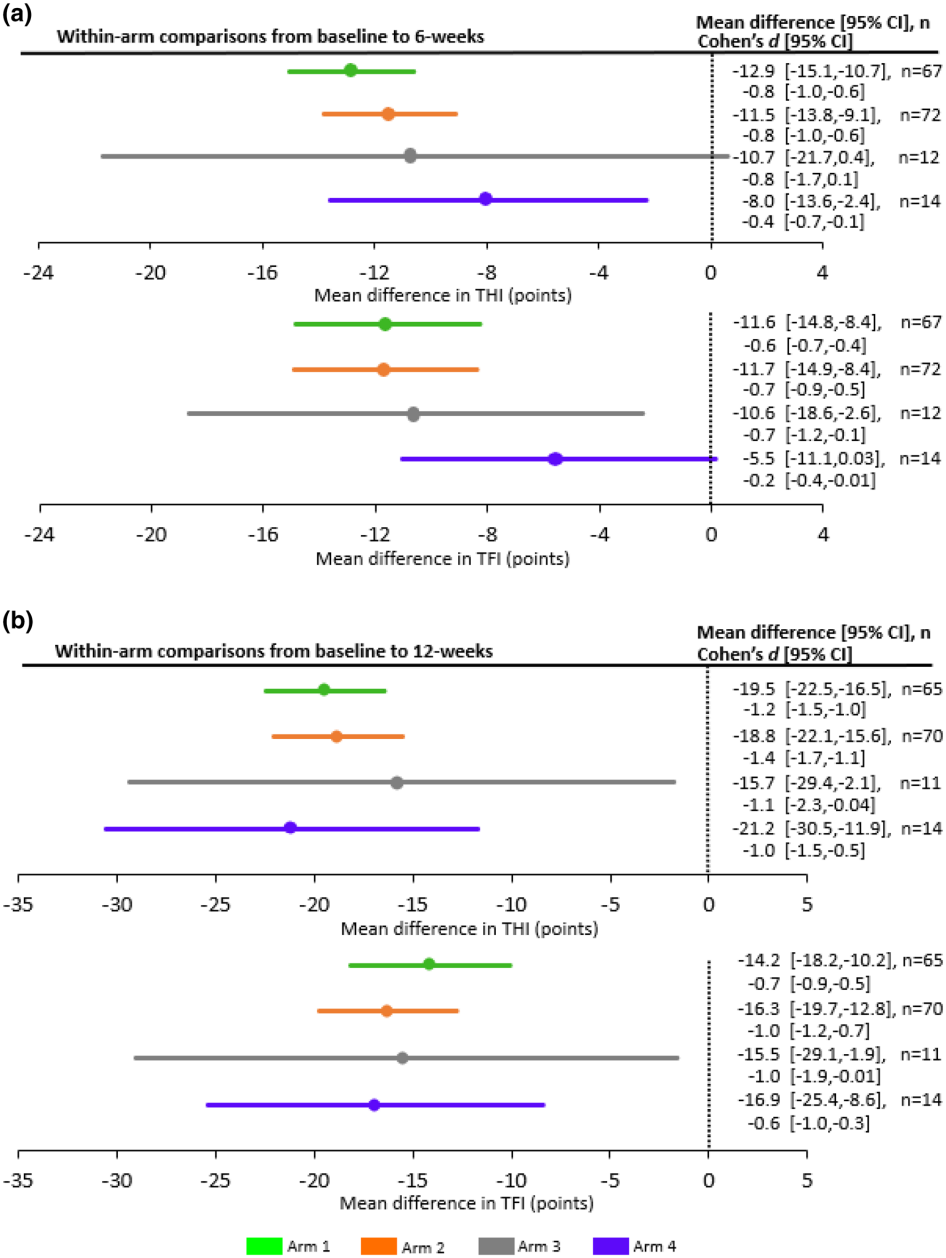
Within-arm analyses for the four different arms at 6-weeks and at 12-weeks. (**a**) The mean difference in THI or TFI score across participants for each arm from baseline to 6-week assessment are plotted with the ± 95% CI. Within-arm analyses were based on a per-protocol analysis that included treatment-compliant participants (≥ 18 h treatment at 6-weeks) with baseline and 6-week scores (note that the baseline score corresponds to the average of the scores at screening and enrollment visits). Within-arm analyses for all arms, except for arm 3 for THI and arm 4 for TFI, showed a statistically significant reduction in THI or TFI score (i.e., improvement in tinnitus symptom severity) based on paired two-tailed t-tests (*P* < 0.05; 95% CI does not overlap the vertical line at zero). Cohen’s *d* effect size and 95% CI are also listed for each arm for THI and TFI. Parameter settings (see Supplementary Table S1 for details): arm 1—PS1 (synchronized bimodal stimulation, includes pure tones and background noise stimuli); arm 2—PS6 (includes only pure tones with no background noise stimuli and with varied interstimulus delays in the range of 700–800 ms); arm 3—PS7 (same as PS6 except four tones presented simultaneously over a larger frequency range); arm 4—PS9 (same sound stimuli as in PS6 but no tongue stimulation). (**b**) Similar to (**a**) except data is presented from baseline to 12-week assessment for treatment-compliant participants (≥ 36 h treatment at 12-weeks). Parameter settings (see Supplementary Table S1 for details): arm 1—PS1 changed to PS4 (includes pure tones and background noise stimuli and with varied interstimulus delays in the range of 0–30 ms); arm 2—PS6 changed to PS10 (includes only wideband noise stimuli without pure tones and with varied interstimulus delays in the range of 0–30 ms); arm 3—PS7 changed to PS4 (includes pure tones and background noise stimuli and with varied interstimulus delays in the range of 0–30 ms); arm 4—PS9 changed to PS6 (same sound stimuli as PS9 with addition of tongue stimulation).

Figure 6 presents the within-arm changes in THI and TFI scores from baseline to the 6-week assessment for all four arms. Arm 1 and arm 2 data from Fig. 3 are included again to enable visual comparison of the results across parameter settings. Interestingly, PS7 in arm 3 exhibited variable effects on tinnitus symptoms (i.e., represented by the wide 95% CI bars in Fig. 6a); however, these wider CIs may be attributed to the smaller sample size in arm 3 compared to the other arms that will need to be confirmed in a larger future study. THI and TFI scores for each participant and for each arm are shown in Supplementary Fig. S7. Another interesting observation from Fig. 6a is that arm 4 with only sound stimulation achieves small to moderate improvements in tinnitus symptoms with an average reduction of 8.0 points for THI and 5.5 points for TFI (small Cohen’s *d* effect sizes of − 0.2 to − 0.4). This contrasts with the larger mean reduction of about 12 points for THI and about 12 points for TFI in arm 1 and arm 2 with bimodal stimulation (Cohen’s *d* of − 0.6 to − 0.8, corresponding to moderate to large effect sizes). These initial data support the potential criticality of combining tongue stimulation with sound to drive additional therapeutic benefit for tinnitus.

**Figure 7:**
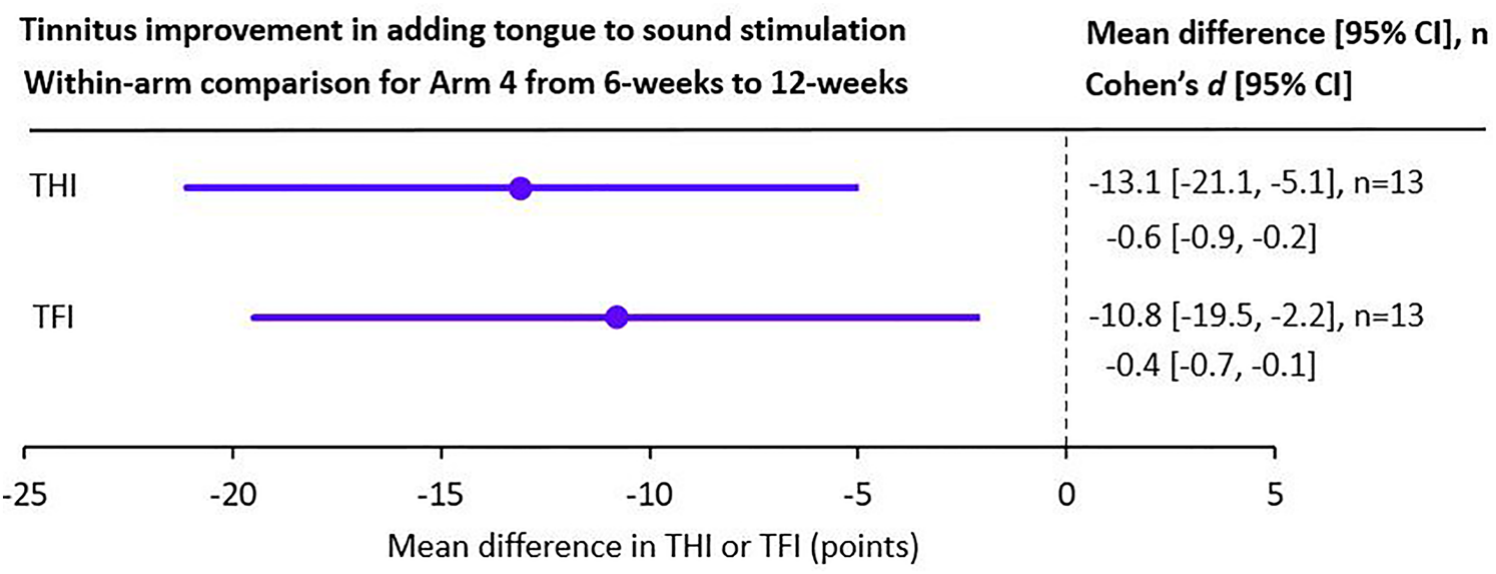
Changes in tinnitus symptom severity for arm 4 between interim and final visits after adding tongue stimulation to sound-only stimulation. Mean change in THI or TFI and 95% CIs are plotted between 6-week (interim) and 12-week (final) visits for all participants who were compliant to treatment (defined as ≥ 18 h at interim and ≥ 36 h at final). There is a significant improvement in tinnitus symptom severity after adding tongue stimulation (PS6) to sound-only stimulation (PS9), supporting the criticality of combining tongue stimulation with sound to drive additional therapeutic benefit for tinnitus. Post-hoc statistical analysis was based on a paired two-tailed t-test (*P* < 0.05; 95% CI does not overlap the vertical line at zero; *P* = 0.0039 for THI and *P* = 0.0180 for TFI). Cohen’s *d* effect size and 95% CI are also listed for THI and TFI on the right side of the figure. Note that n is equal to 13 instead of 14 as in Fig. 6 for arm 4 because compliance criteria of both 18 h and 36 h (rather than just one of them) must be satisfied by each participant to be included in the analysis.

As with arm 1 and arm 2, the stimulation setting was changed between the first and second halves of the 12-week treatment period for arm 3 and arm 4. For arm 3, changing PS7 to PS4 did not reduce the variability in outcomes observed during the first 6 weeks of treatment, though there was a further reduction in mean THI and TFI scores (about − 11 points down to about − 16 points; Fig. 6b). For arm 4, following the 6 weeks of sound-only stimulation with 6 weeks of bimodal stimulation (PS9 to PS6) resulted in an equivalent 12-week improvement as occurred with the other arms (− 21.2 points for THI and − 16.9 points for TFI). In particular, the addition of tongue stimulation to sound stimulation led to substantial improvements in Cohen’s *d* from − 0.4 to − 1.0 for THI and − 0.2 to − 0.6 for TFI (Fig. 6). Figure 7 further shows significant within-arm improvements in THI or TFI between interim and final visits after adding tongue stimulation to sound stimulation. These preliminary results support the criticality of combining tongue stimulation with sound in bimodal stimulation to drive additional benefit for tinnitus, which can be validated in a proceeding confirmatory study.

**Figure 8:**
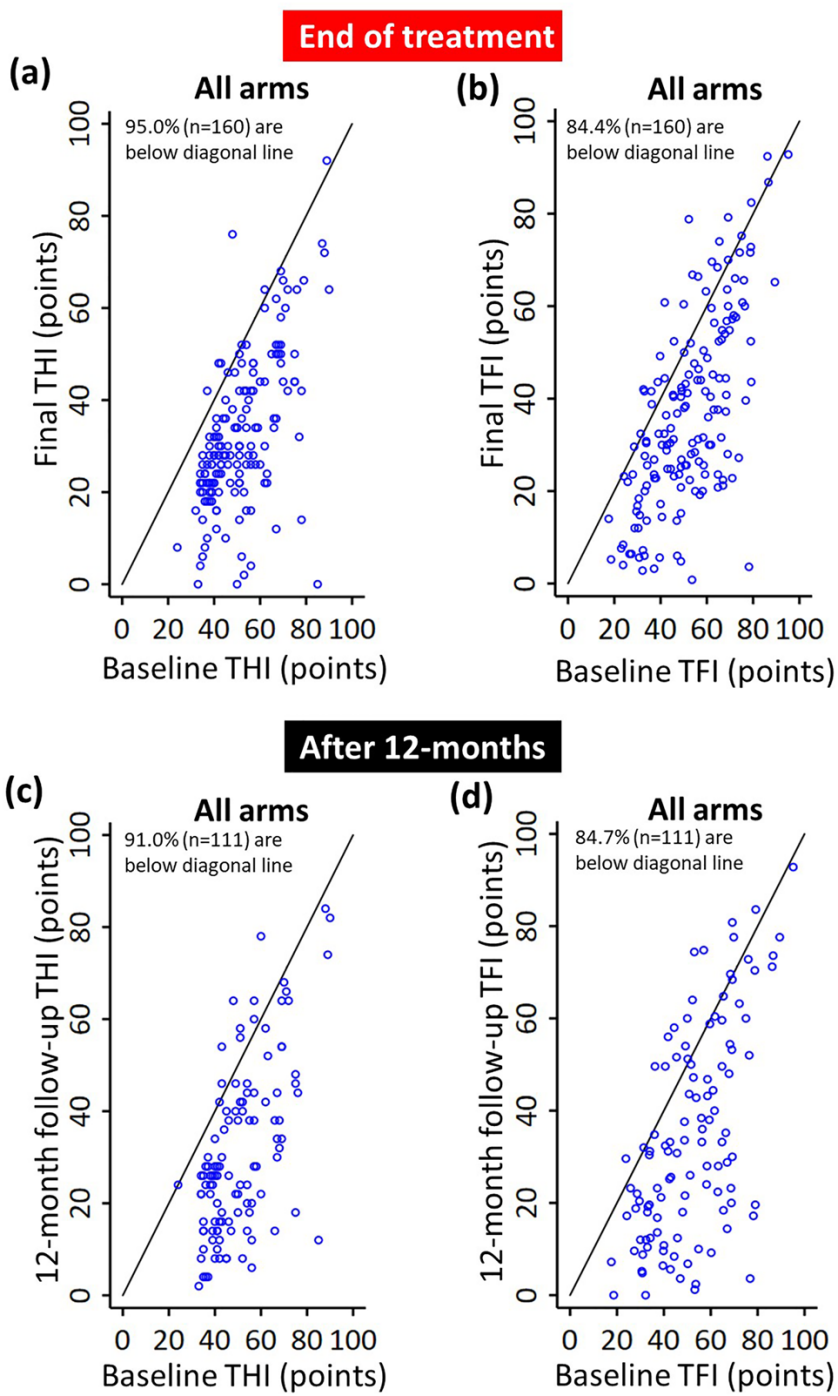
Long-term clinical efficacy of bimodal neuromodulation. (**a,b**) Scatter plots of THI or TFI scores are shown that include each treatment-compliant individual at baseline versus end of treatment (12-week final visit) for all arms pooled together to represent bimodal neuromodulation in general. (**c,d**) Scatter plots of THI or TFI scores are shown that include each treatment-compliant individual at baseline versus 12-month post-treatment visit for all arms pooled together. All individuals who completed at least the assessments displayed in each figure were included to maximize the total number of subjects plotted in each figure, leading to a large sample size for each plot. Compliance to treatment is defined as ≥ 36 h treatment at final. Data points are jittered for visibility.

The individual THI and TFI scores for each participant across all four arms are shown in Supplementary Fig. S8 and are consistent with the findings and interpretations related to Fig. 6b. Across all arms and at the end of treatment, the Cohen’s *d* effect sizes ranged from − 1.0 to − 1.4 for THI and − 0.6 to − 1.0 for TFI (listed in Fig. 6b). Furthermore, 95.0% of treatment-compliant participants (n = 160) had improvements in THI score and 84.4% of treatment-compliant participants (n = 160) had improvements in TFI score at the end of treatment (Fig. 8a,b). This high percentage of responders was sustained out to 12 months after treatment ended, with 91.0% and 84.7% exhibiting improvements in THI and TFI, respectively (Fig. 8c,d; n = 111). Overall, these findings demonstrate that different bimodal stimulus components and parameters that are adjusted over time achieve significant and long-term improvements in tinnitus symptoms in a large percentage of treatment-compliant participants using the Lenire neuromodulation device.

### Lenire treatment achieves strong benefit-to-risk profile and high acceptability of device

A contract research organization (Covance by Labcorp, Durham, NC, USA) assisted with closing out the TENT-A2 study. Adverse event type, seriousness, relationship to study device, and whether or not an adverse event was anticipated was adjudicated by an independent device safety physician employed by Covance by Labcorp with support from the investigators and Medical Review Board. An adverse event (AE) or a serious adverse event (SAE) was classified in line with ISO 14155:2011/MEDDEV2.7, and grouped into three relatedness categories: device related, probably device related, and possibly device related (definitions are provided in “Methods”).

In this study, there were no treatment-related SAEs. We list the AEs potentially related to treatment across all arms for this study in Table 2. There was a total of 156 treatment-related AEs, with 13 classified as related to the device, 58 being probably related to the device, and 85 being possibly related to the device. Of the different AEs, the most common was an increase in tinnitus symptoms, with a total of 79 AEs. This number corresponds to 71 unique individuals (i.e., a single participant could have more than one AE for increased tinnitus symptoms that occurred at different stages during and after treatment). Table 3 further classifies the increased tinnitus AEs based on the arm and stage of treatment (based on a total of 71 cases that occurred during treatment across the two stages listed). For the first 6-week period of treatment from enrollment to interim, there was no significant difference in rates across arms for increased tinnitus AEs (*P* > 0.05; Fisher’s exact test). There was also no significant difference in rates across arms during treatment from interim to final (*P* > 0.05). Bimodal stimulation did not result in any noticeable differences in rate of increased tinnitus AEs compared to the sound-only condition (first row of data in Table 3), suggesting that this type of AE is associated with the sound component rather than the tongue component. In a recent large-scale study characterizing the natural history of tinnitus in adults, 9% of participants (94 out of 1039) reported a worsening of their tinnitus^24^; thus, a proportion of these increased tinnitus AEs are likely due to natural variation in tinnitus symptoms over time. Increase in tinnitus was self-reported by participants during compliance calls or directly to the investigation team either at a clinic visit or in between clinic visits if participants experienced that their tinnitus was louder or more bothersome. There were also several cases of different types of AEs involving the mouth or tongue area. Any unanticipated AEs that were not described in the clinical study documents or expected from the previous TENT-A1 study^11^ are listed in Table 4. Of the 156 treatment-related AEs, 135 AEs were resolved with the participants by the end of the study. The remaining 21 AEs correspond to 18 unique individuals where 12 of them were referred to the appropriate medical professional during or at the end of the study. Six of the 18 individuals could no longer be contacted during the study.

**Table 2:** Safety data (AEs) recorded throughout the study. There were no treatment related SAEs.

Device related	# Events	Probably device related	# Events	Possibly device related	# Events
**Total**	**13**	**Total**	**58**	**Total**	**85**
Increased tinnitus	7	Increased tinnitus	32	Increased tinnitus	40
Headache	4	Tinnitus pitch change	4	Cold sore	12
Unanticipated AE	2	Sore tongue	3	Headache	7
		Abnormal sensation on teeth	2	Sensitive tongue	4
		Sensitive tongue	2	Mouth ulcer	3
		Tongue irritation	2	Tinnitus pitch change	3
		Burning sensation on tongue	1	Blister on tongue	2
		Dizziness	1	Blister in oral cavity	1
		Fluctuating tinnitus	1	Ear pain	1
		Irritation on lips	1	Fluctuating tinnitus	1
		Metallic taste	1	Hearing loss	1
		Numbness on tongue	1	Pulsatile sound in ear	1
		Pain in the mouth	1	Pulsatile tinnitus	1
		Pulsatile sound in ear	1	Unanticipated AE	8
		Sensitivity on mouth area	1		
		Sensitivity to metal dental filling	1		
		Unanticipated AE	3

**Table 3:** Percentage of participants with increased tinnitus AE for each arm and stage of treatment relating to AEs listed in Table 2. P-values were calculated for comparing across arms using Fisher's exact test. There were 71 total cases for the two stages listed (8 additional cases occurred outside of these two stages).

	Arm 1 (n=79)	Arm 2 (n=80)	Arm 3 (n=17)	Arm 4 (n=15)	p-value
Enrollment to interim	34.2% (bimodal)	21.3% (bimodal)	35.3% (bimodal)	26.7% (sound only)	0.270
Interim to final	3.8% (bimodal)	13.8% (bimodal)	11.8% (bimodal)	6.7% (bimodal)	0.128

**Table 4:** Unanticipated treatment related AEs listed in Table 2.

Device related	Probably device related	Possibly device related
Jaw pain	Fuzzy head	Ear discomfort (n=3)
Sensitivity of teeth	Sensitivity of teeth	Anxiety
	Change in tinnitus sound	Eyelid twitching
		Facial pain
		Mouth dryness
		Pimple on pinna

**Figure 9:**
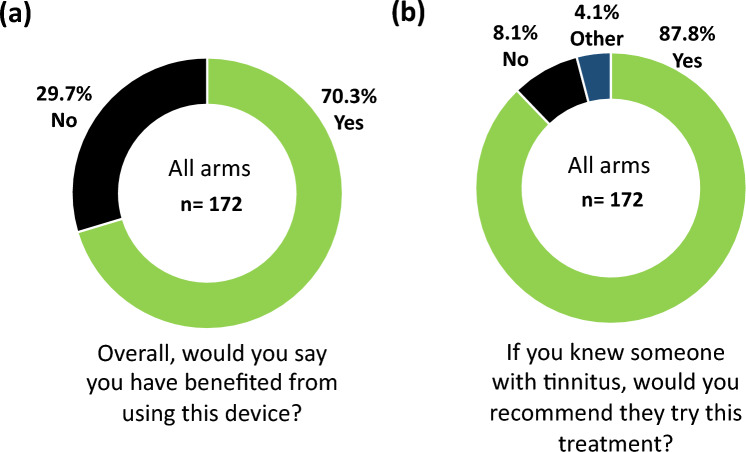
Satisfaction rates in using treatment device. Two questions relating to the participant’s satisfaction (**a**) or acceptability (**b**) of the treatment device were asked at the final visit when the treatment ended, in which the percentage of YES or NO responses are shown. The “other” category refers to cases where the participant could not answer YES or NO or did not feel comfortable in committing to an answer.

Overall, the treatment proved to be safe and well tolerated with no SAEs and a high satisfaction rate across a large cohort of participants. A high percentage of participants used the device for at least the minimum compliance of 18 h over the first 6 weeks of treatment (86.4% of 191 across arms) and a compliance of at least 36 h over the entire 12-week treatment period (83.8% of 191; Supplementary Fig. S2). At the end of treatment, participants were asked “Overall, would you say you have benefitted from using this device?” Out of 172 responses, 70.3% indicated “Yes” (121 responders; Fig. 9a). They were also asked “If you knew someone with tinnitus, would you recommend they try this treatment?” Out of 172 responses, 87.8% indicated “Yes” (151 recommenders; Fig. 9b). Not all 191 participants were available to answer these two questions (i.e., no responses from 19 individuals), in which some of them may not have benefited from the treatment. Even if assuming that all 19 individuals were non-responders of treatment, this worst-case scenario would still result in 63.4% (121 out of 191) benefiting from treatment, along with 79.1% (151 out of 191) having a positive enough experience with the Lenire device to recommend it to others suffering from tinnitus. These high compliance and satisfaction rates, when compared to the reported AEs, support a strong benefit-to-risk profile for this medical device treatment for tinnitus.

## Discussion

The TENT-A2 study was designed to investigate the effects of different sound and tongue stimuli on reducing tinnitus symptom severity over time. The study was statistically powered to compare two treatment arms (arm 1 and arm 2) to determine whether background wideband noise was necessary for bimodal treatment efficacy. An additional objective was to determine whether adjusting the parameter settings at the midpoint of the 12-week treatment regimen could drive additional therapeutic benefits. In the TENT-A2 study, we observed significant improvements in tinnitus symptoms for both arms within 6 weeks of initiating treatment. In arm 1 there was a mean reduction in THI and TFI score of 12.9 and 11.6 points, respectively, and in arm 2 there was a mean reduction of 11.5 and 11.7 points, respectively (Fig. 6a; Cohen’s *d* of − 0.6 to − 0.8, corresponding with moderate to large effect sizes^23^). There was no significant difference in outcomes between arm 1 and arm 2 for THI or TFI. Although this null difference does not reveal which parameter setting is superior (i.e., arm 1 versus arm 2), the within-arm results confirm that the background noise component is not necessary for achieving significant reductions in tinnitus symptom severity with bimodal stimulation.

One major discovery from the TENT-A2 study is that changing the parameter settings during the second 6-week period of the 12-week treatment, regardless of the stimulus features within the first 6 weeks of treatment, significantly drives additional improvements in tinnitus symptoms (Fig. 5). At the end of treatment, Cohen’s *d* effect sizes could reach as large as − 1.4 for THI and − 1.0 for TFI (Fig. 6b), which are considered large effect sizes^23^. These results confirm that adjusting parameter settings over time can provide additional therapeutic benefit and opens up new opportunities for enhancing clinical outcomes with the Lenire device. Furthermore, the results confirm that these enhanced therapeutic benefits can be sustained out to 12 months after the end of treatment (Fig. 8).

Another objective of the TENT-A2 study was to reproduce the safety, efficacy, treatment compliance and patient satisfaction results from the earlier TENT-A1 study^11^. While there was a similarly high compliance rate of about 84% for the 12-week treatment for both studies, we observed better efficacy values in this TENT-A2 study compared to the previous TENT-A1 study. We attribute this finding to the additional therapeutic benefit achieved by adjusting parameter settings midway through the treatment period. We believe that this new stimulation strategy overcomes the treatment habituation effects observed in the TENT-A1 study, where there were diminishing improvements in the second 6-weeks using the same stimulation setting for the full 12-week treatment period. The mean reduction in THI and TFI scores ranged from 14.2 to 21.2 points in the TENT-A2 study, whereas THI and TFI scores ranged from 13.2 to 14.6 points in the TENT-A1 study. In terms of percentage of responders, 95.0% of treatment-compliant participants (n = 160) had improvements in THI score and 84.4% of treatment-compliant participants (n = 160) had improvements in TFI score at the end of treatment in the TENT-A2 study. For the TENT-A1 study, 86.2% of treatment-compliant participants (n = 260) had improvements in THI score and 81.3% of treatment-compliant participants (n = 256) had improvements in TFI score at the end of treatment. Furthermore, 70.3% of participants in the TENT-A2 study reported a benefit from bimodal treatment compared to the 66.5% of participants in the TENT-A1 study; also, 87.8% would recommend the treatment to other tinnitus sufferers in the TENT-A2 study compared to the 77.8% in the TENT-A1 study. Both studies were consistent in terms of the safety profile of the Lenire device, in which the highest percentage of AEs were associated with increased tinnitus during or after the treatment period. There were several AEs in the TENT-A2 study that were unanticipated (see Table 4), but they were not serious AEs and the Lenire device still exhibited a strong benefit-to-risk profile as was observed in the previous TENT-A1 study.

Two additional arms (arm 3 and arm 4) were included in the study to further explore the effects of different stimulus features on tinnitus treatment. Arm 3 included a different type of sound stimulus than used in the other arms, in which four inharmonic-related tones were presented simultaneously instead of single tones or dual harmonically-related tones. Arm 2 included what we view as the minimalist setting (PS6) for bimodal neuromodulation that includes tongue stimulation with only pure tones (no background noise component). Arm 4 further dissected out the contribution of bimodal stimulus features on tinnitus treatment by removing tongue stimulation from PS6 (referred to as PS9). The sound component in PS6 and PS9 were identical. There were two main observations for the results from arm 3 and arm 4. First, the use of complex tone stimuli appeared to introduce wide variability in outcomes in tinnitus symptoms, in which some participants exhibited large improvements while others experienced minimal improvements in THI and TFI scores. This finding suggests that the type of tone stimuli may lead to different outcomes across tinnitus patients. Second, sound-only stimulation (without tongue stimulation) resulted in small improvements in tinnitus symptoms relative to what was observed for bimodal neuromodulation (Cohen’s *d* of − 0.2 to − 0.4 for sound alone compared to − 0.6 to − 1.0 for bimodal stimulation). Adding tongue stimulation to sound-only stimulation achieved additional improvements in tinnitus symptoms, as shown in Fig. 7. These results suggest that tongue stimulation plays a critical role in driving significant therapeutic effects for tinnitus treatment.

The overall findings from this TENT-A2 study indicate that a diverse range of electrical and sound stimulus combinations can significantly improve tinnitus symptoms and aligns with findings from previous animal and human research on bimodal neuromodulation^7–16^. Animal studies have shown that sound stimulation paired with electrical stimulation of different body locations (or related nerves), such as the face, neck, ear, tongue, back and limbs can all drive extensive neural plasticity across the auditory pathway, including in the cochlear nucleus, inferior colliculus and auditory cortex^7,8,10,13–16^. Both pure tone bursts and wideband noise bursts can be effective at driving significant auditory plasticity with bimodal neuromodulation. There is also increasing evidence that a diverse range of bimodal stimulation patterns can drive greater plasticity within the auditory system and lead to larger reductions in tinnitus symptoms compared to sound-only stimulation^7,8,13,16^. For example, one study in a tinnitus animal model has shown that bimodal stimulation with single pure tones (e.g., 8 kHz) paired with electrical stimulation of the neck region with a specific interstimulus delay of 5 ms elicits neural plasticity and improvements in behavior associated with tinnitus to a larger extent than achieved with sound stimulation alone^7^. Another study in animals demonstrated that bimodal stimulation with wideband noise paired with electrical stimulation of the tongue, neck or mastoid region with different interstimulus delays elicits neural plasticity within the auditory cortex or inferior colliculus that is about double of what is achieved with sound stimulation alone^8^. These animal results are consistent with recent findings in humans using bimodal neuromodulation to treat tinnitus. In 20 human participants, bimodal neuromodulation with a complex sound stimulus (i.e., frequency spectrum matched to the tinnitus percept instead of a single pure tone) paired with electrical stimulation of the neck or face region reduced tinnitus symptoms by about 2 to 3 times more than sound stimulation alone^7^. In the current TENT-A2 study, bimodal stimulation with several different sound stimuli paired with tongue stimulation with different interstimulus delays also drove reductions in tinnitus symptoms that was about double of what was observed with sound stimulation alone. In summary, these findings across animal and human studies provide consistent evidence that multiple types of sound stimuli paired with electrical stimulation of different body regions using a range of interstimulus delays can all be effective at treating tinnitus that exhibit greater auditory plasticity and improvements in tinnitus symptoms than achieved with sound stimulation alone.

## Methods

### Study design

The study was a prospective, randomized, double-blind, four-arm parallel study to determine the contribution of different features of bimodal stimulation on clinical outcome and to investigate the therapeutic effects of changing parameter settings over time for a 12-week tinnitus treatment regimen. The TENT-A2 study was conducted as a follow-up to the earlier TENT-A1 trial^11^. As both trials assessed the same device, with additional parameters sets in TENT-A2 compared to TENT-A1; the study design, recruitment of participants, and statistical methodology of both trials are comparable. The description of methods in the following sections are similar to the published TENT-A1 trial^11^ with additional details on specific procedures applied in the TENT-A2 study.

In TENT-A2, study participants were recruited to the clinical trial using radio advertising and directed to an online eligibility assessment. Eligibility was initially assessed through a set of general pre-screening questions to manage the large number of candidates expected to respond to the advertising^22^. Of these potential participants, 462 signed an informed consent and attended a clinical screening visit. All candidates were assigned a Unique Identifier Code (UIC) to ensure that files and data could be managed in a de-identified manner throughout the study. Of the 462 candidates (Fig. 2), 194 individuals were randomized to the different treatment arms of which 192 of them attended the enrollment visit. One participant was withdrawn at the enrollment visit by the investigators due to a previously undisclosed medical condition. Out of 191, 79 participants were allocated to arm 1 and 80 participants were allocated to arm 2 that are relevant for the primary endpoint analyses.

When participants returned for their enrollment visit, we performed several tinnitus assessments and health evaluations. Each participant was then fitted with a Lenire device (CE-marked Class IIa; Neuromod Devices, Dublin, Ireland) and provided a training session on how to use the device. Participants did not receive any other tinnitus education or counselling during the course of the study. The take-home device was self-administered by the participants with a recommended use of two 30-min sessions per day over a 12-week period. These two sessions could be performed consecutively or at different times during the day. Outcome measures and health evaluations were performed at interim (6-week visit) and at final (12-week visit at end of treatment). Participants returned their devices at the 12-week visit and were invited for post-treatment assessments at Week 18 (6-week follow-up), Week 38 (6-month follow-up) and Week 64 (12-month follow-up). Safety information was collected throughout the study with any adverse events recorded from screening to the 12-month follow-up assessment. There were also two compliance phone calls at Week 3 and Week 9 of the study to remind participants of the recommended use instructions. Compliance to treatment for the primary endpoint analyses was defined as device usage of at least 18 h by the 6-week interim assessment, with an additional treatment compliance criterion of at least 36 h by the 12-week final assessment for other analyses presented in this paper and based on previous publications^11,21^. The study timeline is depicted in Fig. 1b with the number of participants attending and completing assessments for each arm listed in Fig. 2 and Table 1.

For randomization of participants to the four different treatment arms (5:5:1:1), stratification was performed based on four binary categories obtained at screening and using the minimization method^25^, as described in the published protocol paper^21^. These categories include: (1) hyperacusis as defined as having a sound level tolerance (or loudness discomfort level, LDL) that was less than 60 dB sensation level (SL) for a pure tone presented at 500 Hz in either ear (dB SL equals dB HL minus hearing threshold level of sound stimulus); (2) hyperacusis with a LDL less than 70 dB SL; (3) high THI with a score of greater than 56 points; (4) unilateral tinnitus; and (5) participants who do not fall in any of the previous categories. Since we did not incorporate additional clinical criteria and questionnaire data relevant for classifying hyperacusis into the stratification process, this category should be interpreted more strictly as a sound sensitivity condition specific to 500 Hz rather than what is typically considered as hyperacusis. The word “hyperacusis” is still used throughout this paper to remain consistent with the previously published stratification terminology for the clinical study^21^.

Similar to that in TENT-A1^11^, the Lenire device delivered sound wirelessly via Bluetooth headphones while electrical stimulation was delivered to the surface of the tongue using a wired 32-site electrode array (Fig. 1a). The transmission delay via the Bluetooth headphones was extensively characterized and, accordingly, the Lenire device compensated for the timing variations between sound and tongue stimulation. The delay variation was maintained within ±  3 ms, with mean delay variation for each treatment session staying within  ± 2 ms. Electrical stimulation was delivered in the form of biphasic, anodic-leading pulses between approximately 5 and 210 μs duration and with a fixed amplitude. The sound volume range and electrical tongue stimulation intensities were customized to each participant’s sensation thresholds. The participant’s pure-tone audiometric thresholds (250 Hz to 8 kHz) were measured at the screening visit (Supplementary Fig. S1) and subsequently used to configure the intensity of the auditory stimuli to ~ 10 dB SL (i.e., ~ 10 dB above their hearing threshold at each tone frequency). The participant could adjust the default auditory stimulus intensity between -12 dB and + 12 dB during treatment using volume buttons on the controller. For safety reasons, the upper stimulus intensity was limited for participants commensurate with the degree of hearing loss. For those with severe to profound hearing loss, auditory stimuli did not exceed a time-weighted average of about 90 dBA, and for those with normal or mild hearing loss, stimuli did not exceed a time-weighted average of about 70 dBA. Electrical stimulation intensity was configured for each participant by adjusting the intensity from sub-threshold to supra-threshold sensations to a comfortable intensity across different electrodes, which was when the participant could feel sensations on the tongue but below an intensity that was bothersome or painful. This comfortable intensity was used as the calibrated setting, and the participant could adjust the electrical stimulation intensity down to 60% and up to 160% of this calibrated setting using buttons on the controller. The treatment device reverted to the default intensities at the start of each new session.

The sequential stimulus settings utilized in this study for each arm during the first and second 6-week stages of the 12-week treatment period are described in detail in Supplementary Table S1. Briefly, PS1 followed by PS4 was used in arm 1, PS6 followed by PS10 was used in arm 2, PS7 followed by PS4 was used in arm 3, and PS9 followed by PS6 was used in arm 4. PS1 was identical to the parameter setting used in the previous TENT-A1 study^11^ that consisted of tongue stimulation temporally and spatially synchronized with different pure tones with additional background wideband noise. PS4 was similar to PS1, except that there were short delays between sound and tongue stimulation as well as a randomized tone-to-tongue spatial mapping. In TENT-A1, a stimulation strategy similar to PS4 still showed significant improvements in tinnitus symptoms; thus, PS4 provided an alternative stimulation pattern than PS1 that could potentially overcome the habituation effects observed in TENT-A1 during the second 6-weeks of treatment. PS6 consisted of only pure tones paired with tongue stimulation with longer interstimulus delays than used in PS4 and without any background noise component, which was designed to address one of the main objectives of the study as described in the Introduction. PS10 was similar to PS4, except that noise burst stimuli were presented instead of pure tones for the sound component in order to assess if use of only noise stimuli could potentially overcome habituation effects if PS4 was not sufficient. PS7 was similar to PS6 except that four simultaneous tones across a larger frequency range were used for the sound stimulus instead of single or dual tones in order to explore if greater diversity in tonal input could affect clinical outcomes. PS9 consisted of the exact same sound stimuli as in PS6 but without tongue stimulation, which allowed assessment of the effects of the addition of the tongue component on improving tinnitus symptoms.

AEs were documented, and categorized in relation to type, seriousness, relationship to the study device, and whether or not an AE was anticipated. The AEs were classified in line with ISO 14155:2011/MEDDEV2.7 and informed by the US Code of Federal Regulations, Title 21, Volume 8, Section 812 where definitions are absent in ISO14155:2011. These AEs were then further grouped into three relatedness categories. Device related was defined as an AE associated with the investigational device, or with procedures beyond reasonable doubt. Probably device related was defined as having a relationship with the use of the investigational device, or the relationship with procedures, that seems relevant and/or the event cannot be reasonably explained by another cause. Possibly device related was defined as having a relationship with the use of the investigational device or relationship with the procedures that was weak but cannot be ruled out completely. Alternative causes are also possible (e.g., an underlying or concurrent illness/clinical condition and/or an effect of another device, drug, or treatment). Cases where relatedness cannot be assessed, or no information has been obtained should also be classified as possible. Assessments of causality were made by an independent device safety physician.

### Participants

The study recruited participants with chronic subjective tonal tinnitus. Chronic subjective tinnitus is a phantom auditory percept that is attributable to abnormal firing patterns in the brain^1^. Subjective tinnitus differs from objective tinnitus, which is typically associated with sounds generated from vasculature or pulsation anomalies, abnormal muscle contractions or head/jaw movements^1^. For screening, participants who were between the age range of 18 and 70 years and with a tinnitus duration between 3 months and 10 years were included in the study. We recruited participants with THI scores of 38 to 100 points and a Minimum Masking Level (MML) measurement between 20 and 80 dB HL. MML was determined by presenting a wide-band noise at increasing intensities until the sound stimulus masked the participant’s tinnitus. We required that the candidates have a maximum hearing loss of 40 dB HL in the measurement frequencies in the range of 250 Hz to 1 kHz or of 80 dB HL in the measurement frequencies in the range of 2–8 kHz for both ears. To be eligible, participants had to be able to read and understand English and provide informed consent. They were also required to be willing to commit to the full duration of the study.

Candidates were excluded if they had objective tinnitus or somatic tinnitus caused by a head or neck injury, or if their tinnitus was comorbid with a neurological condition as assessed by a medical professional. Abnormal otoscopy or abnormal tympanometry was another exclusion criterion. Further exclusions included candidates with: a hearing aid used within 90 days prior to eligibility assessment, any type of electro-active implantable device, a loudness discomfort level of < 30 dB SL at 500 Hz for either ear, a temporomandibular joint disorder, anxiety determined by a score > 120 out of 160 on the State-Trait Anxiety Inventory (STAI)^26,27^, or moderate to severe dementia as indicated by a score < 20 on the Mini-Mental State Examination (MMSE)^28^. A final set of exclusion criteria based on a medical history taken at the screening assessment were: Meniere’s disease, oral piercings, pregnancy, involvement in medico-legal cases, history of auditory hallucinations, current prescription of a drug for a central nervous system pathology, and previous use of a Neuromod Devices’ product. If deemed as not a suitable candidate by the investigators of the study, a participant could also be excluded for other reasons not listed above.

### Clinical study endpoints

The primary endpoint analyses used the THI for performing within-arm and between-arm comparisons for arm 1 and arm 2 from start of treatment to the 6-week assessment^21^. TFI was also used for within-arm comparisons for arm 1 for the primary endpoint analyses. The THI^17,18^ and TFI^20^ are clinical outcome measures commonly used to assess tinnitus symptom severity^19^. These outcome measures have been used across multiple studies that have supported clinical guidelines for tinnitus interventions^5,29^. The only clinically recommended treatment specifically for tinnitus, cognitive behavioral therapy, has also leveraged these multi-item questionnaires for evidence that it is an effective tinnitus intervention^29^.

The THI predominantly assesses the emotional and functional impact of tinnitus, in which 25 items are scored 4/2/0 on a categorical scale corresponding to yes/sometimes/no. The global score of the THI (i.e., sum of points across all 25 items) has a value from 0 to 100, with a higher score indicating a greater negative impact of tinnitus. Relating to the inclusion and exclusion criteria, participants can be enrolled if they have a THI score of 38–100 at the screening visit, which includes moderate (38–56), severe (58–76), and catastrophic (78–100) groups. Participants are excluded if they are in the no/slight handicap (0–16) or mild (18–36) group; thus, those who are less likely to seek Lenire treatment in the real-world clinical setting. There were 62.3%, 30.4%, and 7.3% in the moderate, severe, and catastrophic group, respectively (out of 191 enrolled participants).

The TFI assesses a range of tinnitus-related functional complaints experienced over the week prior to assessment. Each of the 25 items is assessed on an 11-point Likert scale, and the sum of the scores is normalized to give a global score from 0 to 100, with a higher score indicating a greater negative impact of tinnitus.

An additional efficacy endpoint of the study was to determine the therapeutic effects of changing the stimulation parameters over time. The study was specifically designed to assess this endpoint in which one parameter setting was provided to each participant during the first half of treatment and a different parameter setting was provided during the second half of treatment (Fig. 1). THI and TFI scores could then be compared across baseline, interim and final visits to confirm if adjusting stimulus settings over time could drive additional therapeutic benefit during the treatment period.

### Statistical analyses

The primary endpoints and analyses of the study are described in a previously published protocol paper^21^. Primary efficacy analyses included within-arm changes in THI and TFI scores in the full cohort of participants from baseline (average scores at screening and enrollment) to interim (initial 6 weeks of treatment) for arm 1. To account for multiple comparisons, the a priori significance level (alpha) of 0.05 was distributed across several statistical tests, in which these within-arm comparisons were allocated an adjusted significance level of 0.0025. For the primary efficacy analyses, we also performed a between-arm comparison for arm 1 and arm 2 for the changes in THI scores from enrollment to interim with an adjusted significance level of 0.0275. Only THI, which is the most widely implemented tinnitus questionnaire, was used for the between-arm comparison to ensure sufficient allocation of alpha level across required comparisons to achieve the objectives of the study. Both THI and TFI were used for the within-arm comparisons because the alpha level allocation was a small enough portion that comparisons could still be implemented for both outcome measures. The remaining portion of the significance level was allocated to other statistical comparisons for subtyping analyses that will be presented in a subsequent paper and is outside the scope of this paper. Within-arm analyses for an additional endpoint were also performed to compare THI and TFI scores at different time points and assess if greater therapeutic effects are achieved by adjusting parameter settings midway through the treatment period. To account for multiple comparisons for this additional endpoint, the Bonferroni correction was applied to the statistical analyses. For the within-arm primary efficacy analyses, data from treatment-compliant participants were included. The minimum compliance was pre-specified for this study^21^ as device usage of at least 18 h by the 6-week interim assessment (and at least 36 h by the 12-week final assessment for additional analyses), based on efficacy data for the Lenire treatment in a previous large-scale randomized study^11^. The Lenire device was configured to allow extraction of data in regard to time and date of usage, duration of electrode contact with the tongue, and intensities of the auditory and tongue stimuli for each treatment session.

The within-arm analyses used a per-protocol analysis that was tested with a paired two-tailed t-test where assumption of normality was confirmed with the Shapiro–Wilk test. The Wilcoxon signed-rank test was performed for cases that did not satisfy the normality condition. Within-arm effect sizes reported in this paper are based on Cohen’s *d* and are calculated as the mean score at end of treatment minus the mean score at baseline, divided by the pooled SD for the two scores. For between-arm analyses, an intention-to-treat analysis was performed with multiple imputation and tested with multiple regression using enrollment scores as a covariate, where normality and equal variance assumptions were confirmed with the Shapiro–Wilk test and Bartlett’s test, respectively. Missing data was handled by using a Markov chain Monte Carlo multiple imputation method^30,31^. For this method, 10 multiple imputed datasets were first generated to fill in missing predictors. This procedure was followed by a second imputation process that estimated the final outcome variable within each imputed dataset. The imputation was based on a multiple regression model with multiple predictor variables^11,21^. Inferences for the between-arm endpoints were evaluated on each imputed data set and the results combined to yield the estimates, confidence intervals and associated significance values.

There were a few deviations to the protocol in which sensitivity analyses confirmed that the primary endpoints were not affected when including or excluding data from participants associated with those deviations (see Supplementary Information S1 for further details).

## Supplementary Information

Supplementary Information.

## Data Availability

The datasets generated and analyzed during the current study are not publicly available due to ethical restrictions [datasets contain pseudonymized special category health data of EU participants and cannot be available for access] but are available from the corresponding author on reasonable request and with appropriate ethics approval.

## Abbreviations

AE: Adverse event
CI: Confidence interval
dB HL: Decibel hearing level
dB SL: Decibel sensation level
FU: Follow-up
Hz: Hertz
ITT: Intention-to-treat
kHz: Kilohertz
LDL: Loudness discomfort level
μs: Microsecond
ms: Millisecond
MMSE: Mini-Mental State Examination
MML: Minimum masking level
PPA: Per-protocol analysis
PS#: Parameter setting #
SAE: Serious adverse event
STAI: State-Trait Anxiety Inventory
TENT: Treatment Evaluation of Neuromodulation for Tinnitus
TFI: Tinnitus Functional Index
THI: Tinnitus Handicap Inventory
