# Author Correction: Different bimodal neuromodulation settings reduce tinnitus symptoms in a large randomized trial

**DOI:** 10.1038/s41598-023-38312-5

**Published:** 2023-07-10

**Authors:** Brendan Conlon, Caroline Hamilton, Emma Meade, Sook Ling Leong, Ciara O Connor, Berthold Langguth, Sven Vanneste, Deborah A. Hall, Stephen Hughes, Hubert H. Lim

**Affiliations:** 1 Neuromod Devices Limited, Dublin, D08 R2YP Ireland; 2 School of Medicine, Trinity College Dublin, Dublin, D02 R590 Ireland; 3 Department of Otolaryngology, St. James’s Hospital, Dublin, D08 NHY1 Ireland; 4 Trinity Institute for Neuroscience and Global Brain Health Institute, School of Psychology, Trinity College Dublin, Dublin, D02 PN40 Ireland; 5 Department of Psychiatry and Psychotherapy, University of Regensburg, 93053 Regensburg, Germany; 6 Interdisciplinary Tinnitus Center of the University of Regensburg, 93053 Regensburg, Germany; 7 Lab for Clinical and Integrative Neuroscience, School of Behavioral and Brain Sciences, The University of Texas at Dallas, Dallas, 75080 USA; 8 National Institute for Health Research Nottingham Biomedical Research Centre, Nottingham, NG7 2UH UK; 9 Hearing Sciences, Mental Health and Clinical Neurosciences, University of Nottingham, Nottingham, NG7 2RD UK; 10 School of Social Sciences, Heriot-Watt University Malaysia, 62200 Wilayah Persekutuan Putrajaya, Malaysia; 11 Department of Otolaryngology-Head and Neck Surgery, University of Minnesota, Minneapolis, 55455 USA; 12 Department of Biomedical Engineering, University of Minnesota, 312 Church Street S.E., NHH 7-105, Minneapolis, MN 55455 USA

Correction to:* Scientifc Reports* 10.1038/s41598-022-13875-x, published online 30 June 2022

The original version of this article contained an error in Supplementary Information file, where in Arm 2 for first 6-week period, range of pure tone bursts was incorrect,

“Range of pure tone bursts (approximately 1kHz to 2kHz)”

now reads:

“Range of pure tone bursts (approximately 0.5kHz to 1kHz)”

The original Article has been corrected.

