# Supplementary material for: Different bimodal neuromodulation settings reduce tinnitus symptoms in a large randomized trial
Source: Sci Rep. 2022 Jun 30;12:10845. doi: 10.1038/s41598-022-13875-x (PMC9246951; doi:10.1038/s41598-022-13875-x)
Supplement: Supplementary file 1 — Supplementary Information. [file 41598_2022_13875_MOESM1_ESM.docx]

**Supplementary Information**

**Deviations to the trial design and sensitivity analyses**

Of the 191 enrolled participants, 3 of them did not meet the eligibility criteria and are considered deviations to the protocol (i.e., one had an MML exceeding 80 dB HL and two had somatic tinnitus relating to a head or neck injury). There were also two participants who did not complete a tympanometry assessment at screening. This assessment was performed at the 12-week visit and no abnormality of the tympanic membrane was detected in these two participants. Analyses were conducted with and without these five participants. The sensitivity analysis demonstrated no significant effect on the primary endpoint findings; thus, all of these participants were included in the analyses presented in this paper.

During the database validation process, there were 7 participants whose audiogram data were identified as being incorrectly inputted into the database at the screening visit, in which their hearing threshold values were supposed to be lower (or higher for one participant) than what was inputted into the database (i.e., by 5 to 30 dB across one to two frequencies in the range of 3000 to 8000 Hz). In these cases, the sound volume was set to be louder (or lower for that one participant) for some frequencies than intended for the Lenire treatment. Analyses were conducted with and without these participants. The sensitivity analysis demonstrated no significant effect on the primary endpoint findings; thus, all of these participants were included in the analyses presented in this paper.

**Supplementary Figure 1.** **Hearing thresholds for enrolled participants**. Data are plotted for each arm or grouped together and presented for left or right ear. Circle represents threshold value for each frequency and participant, solid line corresponds to mean threshold value for each frequency across participants, and shaded region corresponds to standard deviation of threshold values for each frequency across participants. Data points are jittered for visibility. dB HL: decibels in hearing level.


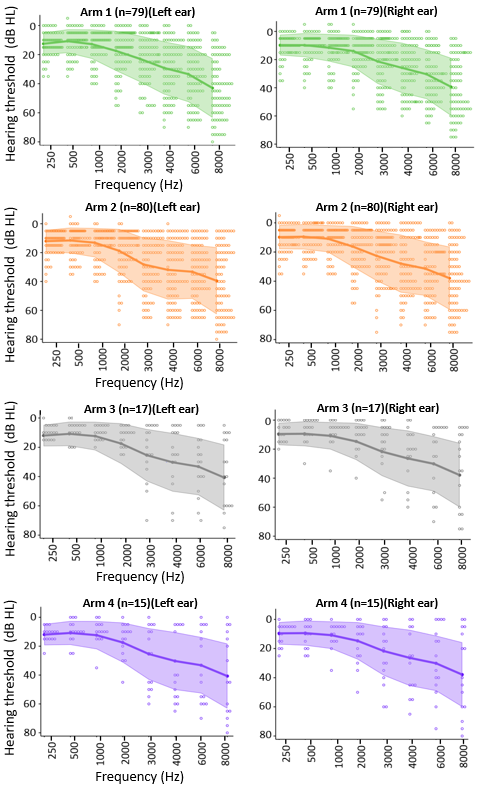


**Supplementary Figure 2.** **Compliance rates in using treatment device. (A)** Numbers and percentages of enrolled participants who achieved the minimum treatment compliance of ≥18 hours over the intended 6-week treatment period. **(B)** Numbers and percentages of enrolled participants who achieved treatment compliance of ≥36 hours over the entire 12-week treatment period. There was no statistically significant difference in compliance rates or numbers of participants between arm 1 and arm 2 (used for the primary endpoint analyses) for either of the treatment periods (*P* > 0.05; Fisher’s exact test).


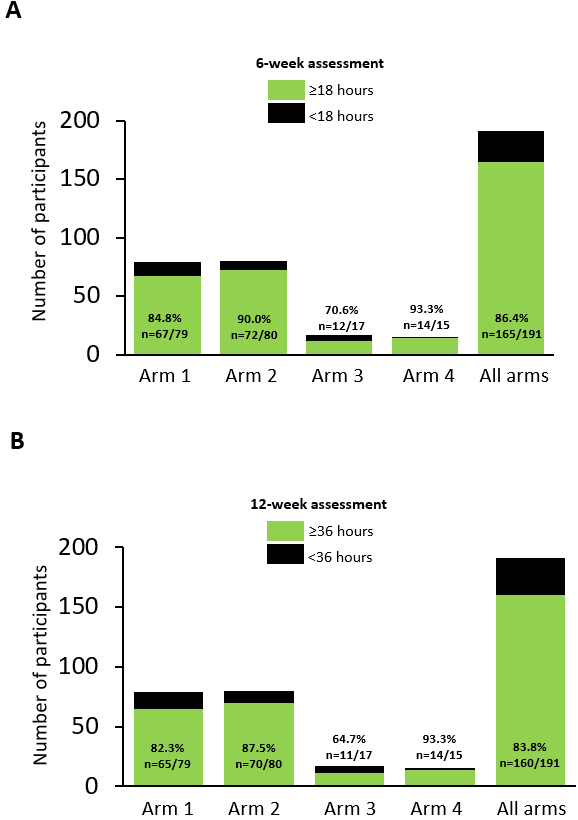


**Supplementary Figure 3.** **Histogram of time (in weeks) when participants attended each visit relative to enrollment.** Treatment began at t = 0 weeks. The participants generally attended each visit at or near the intended time point of 6, 12, 18, 38 and 64 weeks relative to enrollment (see Fig. 1 for study timeline).


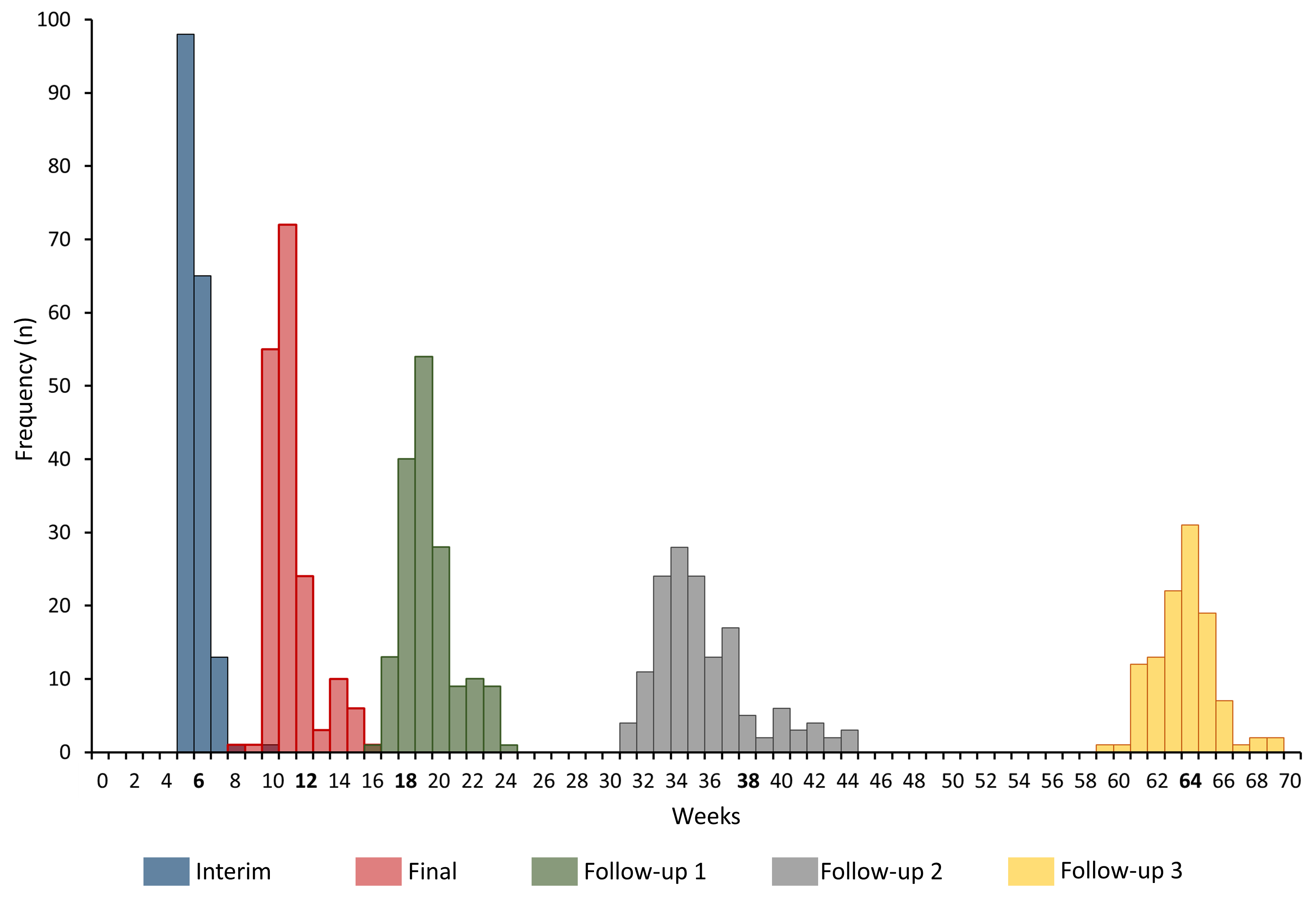


**Supplementary Figure 4.** **Changes in tinnitus symptom severity during and after the 12-week treatment in the TENT-A1 study.** Absolute THI (**A**) or TFI (**B**) scores (distribution of values as violin plots) are presented at different time points during the treatment period at Week 0 (baseline scores plotted at enrollment), Week 6 (interim), Week 12 (final) and Week 64 (12-month post-treatment) for pooled data across arm 1, arm 2 and arm 3 from the TENT-A1 study. Data is included for participants who were treatment-compliant (≥36 hours treatment at final when provided with the same parameter setting for the entire 12-week treatment period) and correspond to the same data shown in Fig. 5 from the previously published paper for the TENT-A1 study^11^. The same parameter setting during the first 6 weeks of treatment was used for the entire 12-week treatment period, unlike in the TENT-A2 study where the parameter settings were changed at the 6-week visit. Asterisks correspond to significant reductions in THI or TFI scores based on a Wilcoxon signed-rank test (*P* < 0.05). Significant P-values accounting for multiple comparisons based on the Bonferroni correction are labeled with asterisks in each plot: (**A**) *P* < 0.00001; (**B**) *P* < 0.00001.


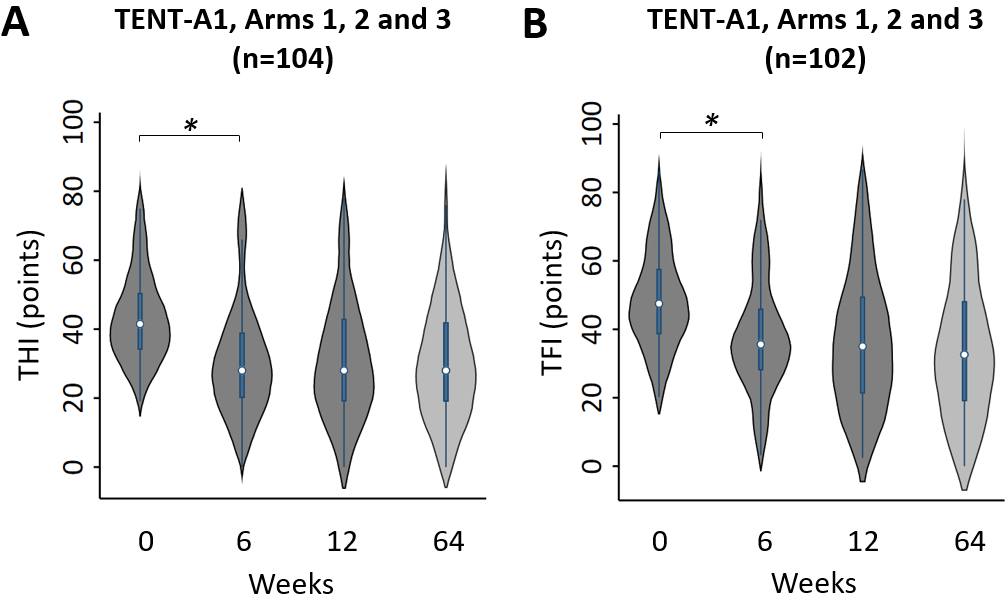


**Supplementary Figure 5.** **Changes in tinnitus symptom severity during and after the 12-week treatment in the TENT-A2 study for arm 1 and arm 2.** Absolute THI (**A, B**) or TFI (**C, D**) scores (distribution of values as violin plots) are presented at different time points during the treatment period at Week 0 (baseline scores plotted at enrollment), Week 6 (interim), Week 12 (final) and Week 64 (12-month post-treatment) for arm 1 and arm 2. Data corresponds to the same data shown in Fig. 5 but separated out for each arm, demonstrating consistent findings with that shown in Fig. 5. P-values are calculated using the Wilcoxon signed-rank test.


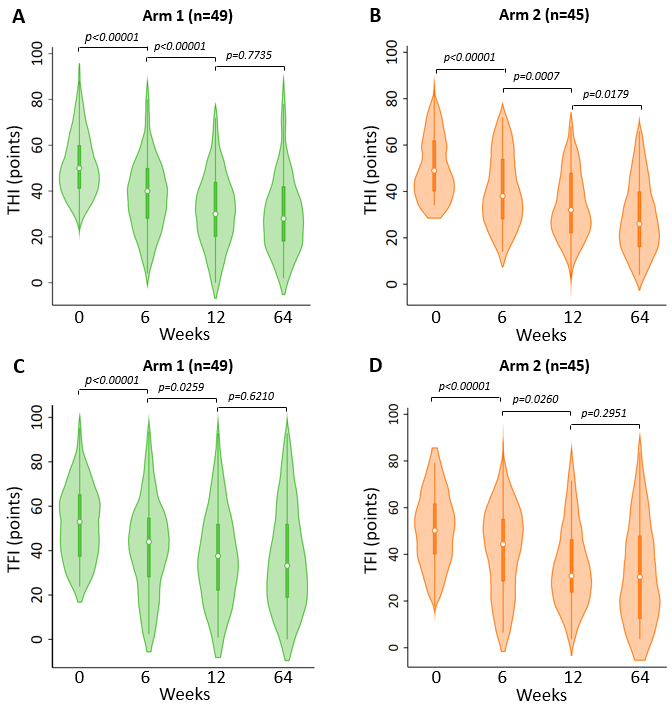


**Supplementary Figure 6.** **Changes in tinnitus symptom severity during and after the 12-week treatment in the TENT-A1 study for arm 1, arm 2, and arm 3.** Absolute THI (**A, B, C**) or TFI (**D, E, F**) scores (distribution of values as violin plots) are presented at different time points during the treatment period at Week 0 (baseline scores plotted at enrollment), Week 6 (interim), Week 12 (final) and Week 64 (12-month post-treatment) for arm 1, arm 2, and arm 3. Data corresponds to the same data shown in Supplementary Fig. 4 but separated out for each arm, demonstrating consistent findings with that shown in Supplementary Fig. 4. P-values are calculated using the Wilcoxon signed-rank test.


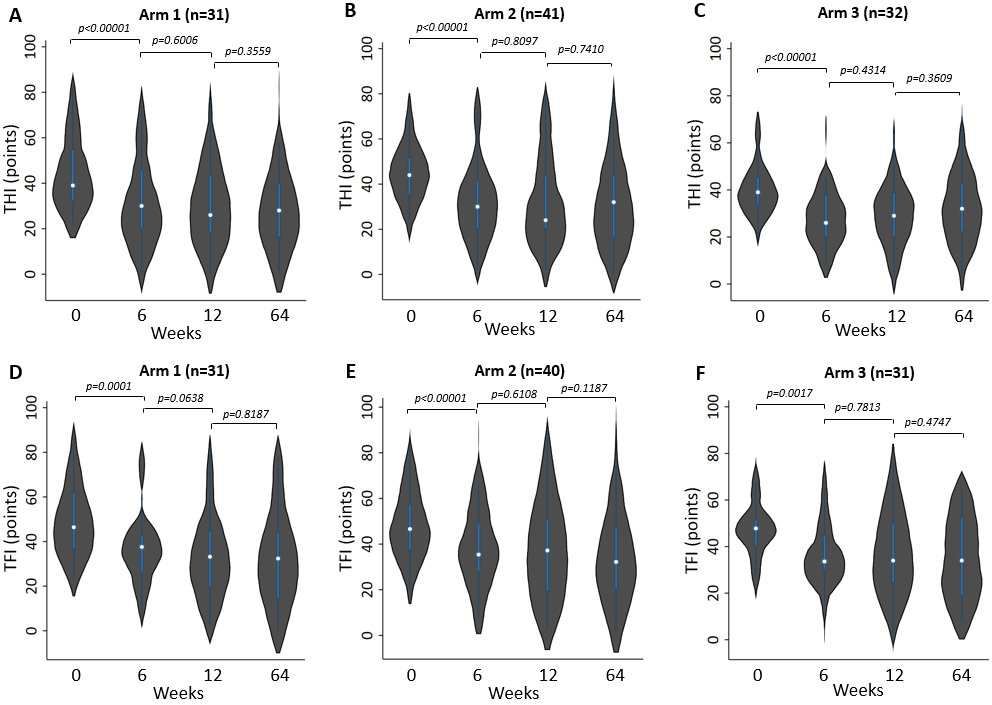


**Supplementary Figure 7.** **Changes in tinnitus symptom severity based on THI or TFI for each participant for each treatment arm at 6-weeks.** Scatter plots of THI scores **(A)** or TFI scores **(B)** are shown that include each treatment-compliant individual for each arm at baseline versus interim (Week 6). Points below the diagonal black line indicate an improvement in tinnitus symptom severity (i.e., a decrease in score) for each outcome measure. All individuals who completed at least the assessments displayed in each figure were included to maximize the total number of subjects included in each figure. Compliance to treatment is defined as ≥18 hours treatment at interim. Data points are jittered for visibility.


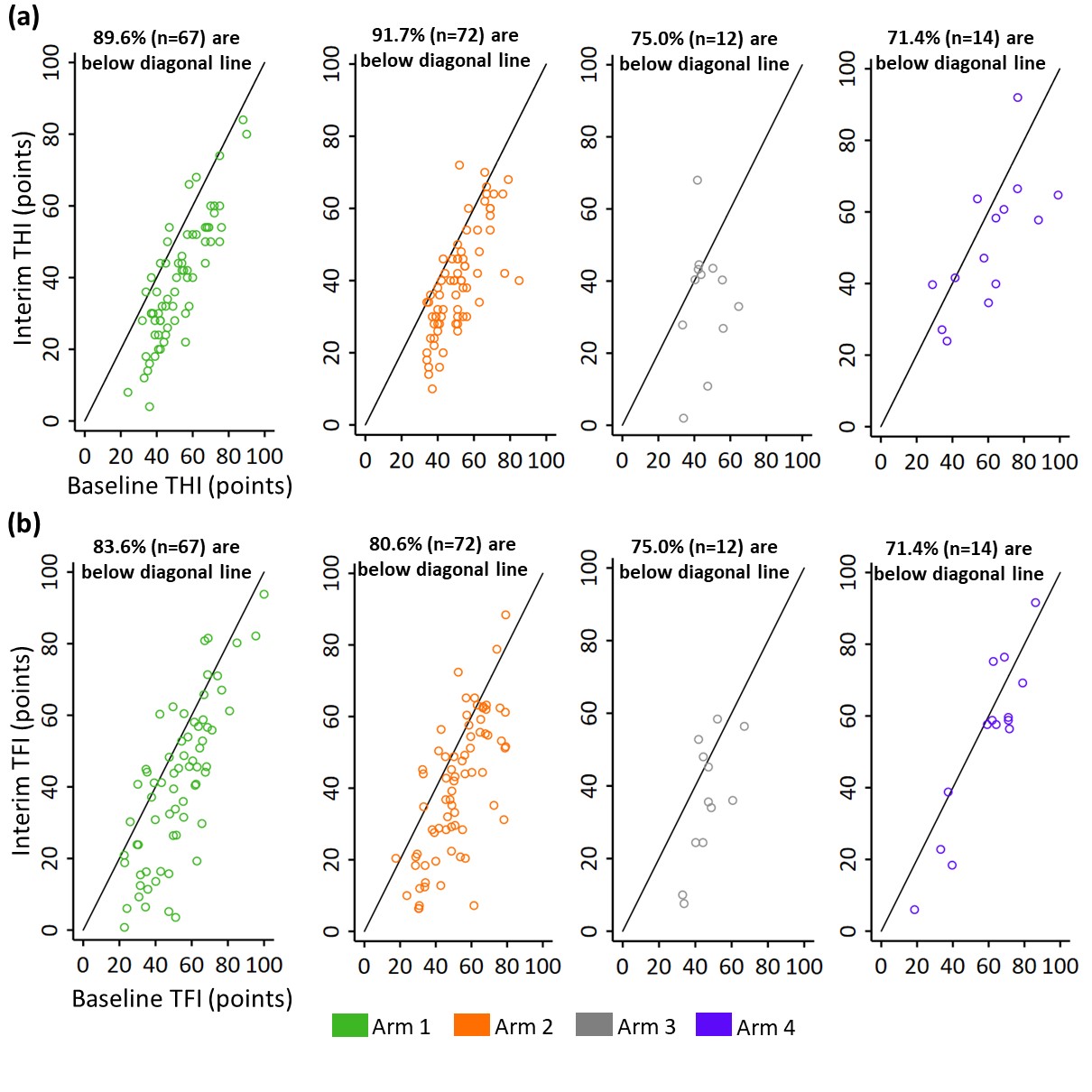


**Supplementary Figure 8.** **Changes in tinnitus symptom severity based on THI or TFI for each participant for each treatment arm at 12-weeks.** Scatter plots of THI scores **(A)** or TFI scores **(B)** are shown that include each treatment-compliant individual for each arm at baseline versus final (Week 12). Points below the diagonal black line indicate an improvement in tinnitus symptom severity (i.e., a decrease in score) for each outcome measure. All individuals who completed at least the assessments displayed in each figure were included to maximize the total number of subjects included in each figure. Compliance to treatment is defined as ≥36 hours treatment at final. Data points are jittered for visibility.

**
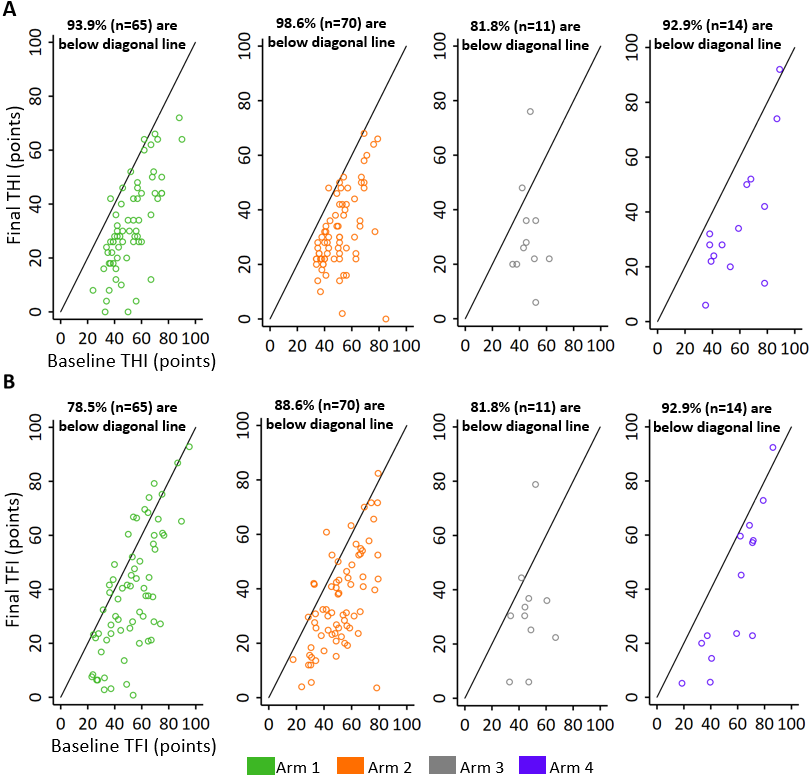
**


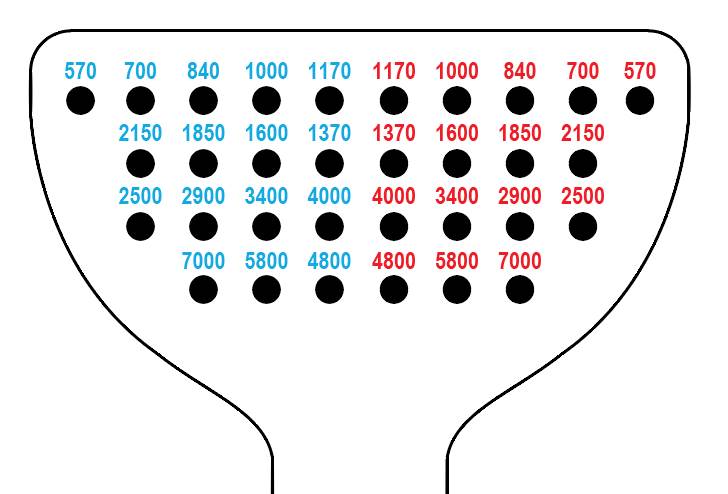
**Supplementary Table 1. Description of parameter setting (PS) for each treatment arm.**

For PS1, tones spanned the sixteen Bark Scale critical bands of hearing where center frequencies of bands are shown in the tongue schematic (left). Each tone was mapped to a specific location on the tongue array, and both sides of the tongue were stimulated symmetrically. A pulse train (5 or 6 pulses) was delivered to the tongue where each pulse had a constant amplitude, but pulse duration was adjusted during fitting to achieve comfortable sensations on the tongue (5 to 210 μs; inter-pulse period of ~3 ms).

| **First 6-week period** | **Arm 1**  **PS1**   - Range of pure tone bursts (approximately 0.5 kHz to 8 kHz) - Presented binaurally (one or two harmonically related tones at a time) - Each tone burst synchronized to tongue stimulus - Tongue stimulus delivered to a specific location on the tongue array according to tone frequency - Background wideband noise   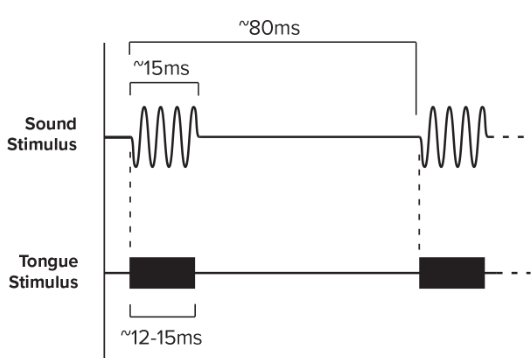 |
| --- | --- |
| **Second 6-week period** | **PS4**  Same as PS1 except for:   - Random short delays between each tone burst and tongue stimulus - Location of stimulation on the tongue randomized across stimuli   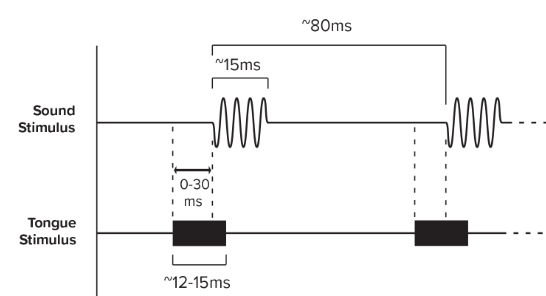 |

| **First 6-week period**  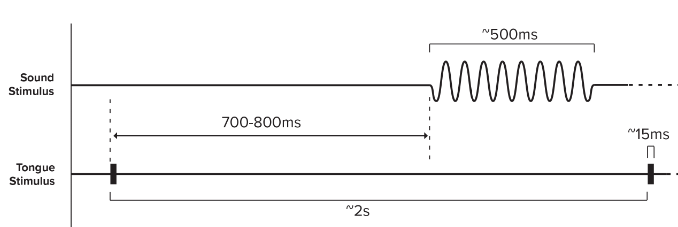 | **Arm 2**  **PS6**   - Range of pure tone bursts (approximately 0.5kHz to 1kHz) - Presented binaurally (one tone at a time) - Random delays between each tone burst and tongue stimulus - Location of stimulation on the tongue randomized across stimuli - No background noise |
| --- | --- |
| **Second 6-week period** | **PS10**  Same as PS4 in arm 1 except for:   - Use of wideband noise bursts instead of pure tone bursts for sound stimuli   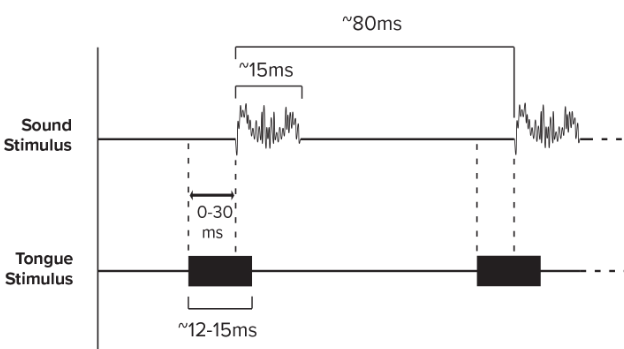 |

| **First 6-week period**  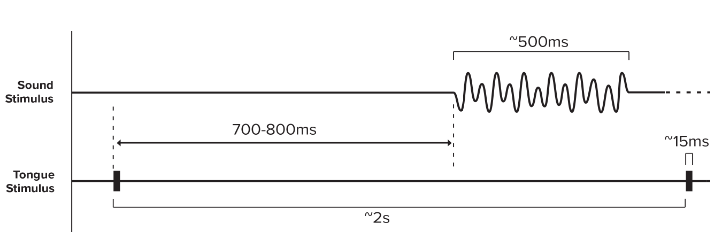 | **Arm 3**  **PS7**  Same as PS6 in arm 2 except for:   - Sound stimuli consisting of multiple simultaneous tones (i.e., four different tones presented each time that are not harmonically related; approximately 0.5 kHz to 7 kHz) |
| --- | --- |
| **Second 6-week period** | **PS4**  Same as PS4 in arm 1 |
| **First 6-week period** | **Arm 4**  **PS9**   - Only sound stimuli that were the same as PS6 in arm 2 |
| **Second 6-week period** | **PS6**  Same as PS6 in arm 2 |
